# Large-scale template-based structural modeling of T-cell receptors with known antigen specificity reveals complementarity features

**DOI:** 10.3389/fimmu.2023.1224969

**Published:** 2023-08-15

**Authors:** Dmitrii S. Shcherbinin, Vadim K. Karnaukhov, Ivan V. Zvyagin, Dmitriy M. Chudakov, Mikhail Shugay

**Affiliations:** ^1^ Institute of Translational Medicine, Center for Precision Genome Editing and Genetic Technologies for Biomedicine, Pirogov Russian National Research Medical University, Moscow, Russia; ^2^ Laboratory of Structural Bioinformatics, Institute of Biomedical Chemistry, Moscow, Russia; ^3^ Center of Life Sciences, Skolkovo Institute of Science and Technology, Moscow, Russia; ^4^ Shemyakin-Ovchinnikov Institute of Bioorganic Chemistry, Russian Academy of Sciences, Moscow, Russia; ^5^ Center of Molecular Medicine, Central European Institute of Technology (CEITEC), Masaryk University, Brno, Czechia

**Keywords:** T-cell receptor, antigen recognition, TCR-peptide-MHC complex, structural modeling, database

## Abstract

**Introduction:**

T-cell receptor (TCR) recognition of foreign peptides presented by the major histocompatibility complex (MHC) initiates the adaptive immune response against pathogens. While a large number of TCR sequences specific to different antigenic peptides are known to date, the structural data describing the conformation and contacting residues for TCR-peptide-MHC complexes is relatively limited. In the present study we aim to extend and analyze the set of available structures by performing highly accurate template-based modeling of these complexes using TCR sequences with known specificity.

**Methods:**

Identification of CDR3 sequences and their further clustering, based on available spatial structures, V- and J-genes of corresponding T-cell receptors, and epitopes, was performed using the VDJdb database. Modeling of the selected CDR3 loops was conducted using a stepwise introduction of single amino acid substitutions to the template PDB structures, followed by optimization of the TCR-peptide-MHC contacting interface using the Rosetta package applications. Statistical analysis and recursive feature elimination procedures were carried out on computed energy values and properties of contacting amino acid residues between CDR3 loops and peptides, using R.

**Results:**

Using the set of 29 complex templates (including a template with SARS-CoV-2 antigen) and 732 specificity records, we built a database of 1585 model structures carrying substitutions in either TCRα or TCRβ chains with some models representing the result of different mutation pathways for the same final structure. This database allowed us to analyze features of amino acid contacts in TCR - peptide interfaces that govern antigen recognition preferences and interpret these interactions in terms of physicochemical properties of interacting residues.

**Conclusion:**

Our results provide a methodology for creating high-quality TCR-peptide-MHC models for antigens of interest that can be utilized to predict TCR specificity.

## 1 Introduction

Specific interaction between T-cell receptors (TCRs) and major histocompatibility complex (MHC)-peptide complexes is a key initiation factor of adaptive immunity-driven physiological processes (1) that happens upon recognition of an infected or antigen-presenting cell by cognate T-cell. TCR molecules are heterodimers encoded by genes formed via the process of V(D)J-rearrangement that ensures the presence of a highly diverse (>10^8^ combinations of TCRα - TCRβ chain pairs) TCR sequences in an individual, required to recognize and target previously unencountered pathogens viewed through the lens of MHC molecules presenting peptides (2, 3). Describing molecular mechanisms governing antigen recognition is therefore the cornerstone of adaptive immunity studies making it possible to predict immune responses to specific antigens, as well as cross-reactivity and selectivity of TCRs in the near future (4). Moreover, present methods, even high-throughput ones such as 10X single-cell sequencing with dCODE dextramers (5), cannot yield enough TCR specificity data to cover the space of all possible TCR:pMHC interactions.

Currently, several databases gathering experimental information about TCR-peptide recognition are available to the research community: VDJdb, IEDB, McPAS-TCR (6–8). These resources store and aggregate data on primary TCR sequences, cognate epitopes and MHC context mined using literature search.

In addition there are more than 280 crystal structures stored in the Protein Data Bank (PDB) that encode the geometry and physical contacts in TCR-peptide-MHC complexes (9, 10). Several computational approaches have been developed for homology-based modeling of individual TCR structures or TCRs in their ternary complex with peptide-MHC molecules they recognize. Promising software tools and approaches in this area include the TCRmodel (11) and TCR-pMHC-models (12) services.

In addition to homology modeling approaches, it has been demonstrated that the AlphaFold software, which is based on deep learning techniques and has revolutionized the field of protein structure prediction, can be employed for *de-novo* modeling of TCR-peptide-MHC complexes. However, the performance of AlphaFold in this context has shown inconsistency. Nonetheless, there is a highly promising pipeline, built upon AlphaFold, that shows potential for enhancing the accuracy of predictions by fine-tuning the modeling parameters, with a particular focus on the specific structural characteristics and regions of TCR-peptide-MHC complexes, such as the CDR3 loops (13).

Recent studies, focused on structural bioinformatics and molecular modeling of TCR-peptide-MHC complexes, can be categorized into two primary areas of research. The first area involves large-scale modeling approaches and mathematical modeling of a significant amount of structural data, while the second area is devoted to a detailed examination of individual complexes and enhancing the precision of binding specificity and affinity inference.

Large-scale attempts to model multiple TCR-peptide-MHC complexes have been carried out in a number of recent studies, most of them were focused on serving as a proof-of-concept and as a benchmark for modeling software, not directly aimed at general applications, such as studying regularities in TCR-peptide-MHC recognition at the residue level or imputing a missing complex structure for a TCR linked to a certain disease. For example, a large database of over 23,000 TCR complex structures with pMHC was generated (14) using template-based approaches for TCR [Repertoire Builder (15), LYRA (16)], peptide - MHC binding prediction (NetMHCpan (17)), and molecular docking (e.g. one can use FlexPepDock (18) to impute peptides in MHC groove), although individual accuracies for these complexes, especially CDR3 loop conformations was not thoroughly validated in each individual case.

Machine learning algorithms have demonstrated their usefulness in predicting TCR-peptide interactions employing high similarity (HS) and random sampling strategies for deep learning models (19) and evaluating binding affinity by training a random forest classifier model using the ATLAS database (20).

On the other hand, it was demonstrated using two contrasting TCR-peptide-MHC test sets that the MMPB/GBSA approach can be effectively applied during the binding affinity calculation. However, the protocol needs to be adjusted based on the similarity of the compared structures (21). Analysis of multiple molecular docking approaches (22) and their corresponding scoring functions has indicated that hydrophobic and electrostatic interactions play important roles in TCR-peptide-MHC recognition. Additionally, *a priori* knowledge of contacting residues of TCR and peptide has been shown to improve modeling of the entire complex, including highly flexible CDR3 loops.

The importance of hydrophobic interactions and pi-stacking forces in TCR contacts with HLA-A*02-restricted peptides, was also analyzed and illustrated using molecular dynamics simulations and binding free energy calculations (23).

Despite the fact that much attention has been paid to the study of SARS-CoV2 recently, only a few COVID-associated TCRs have a resolved TCR-peptide-MHC structure to date (24–27). Having a variety of structures with SARS-CoV-2 epitopes can aid in studying T-cell recognition of infected cells, especially since the number of known COVID-associated TCRs is constantly growing (6).The study of TCR – peptide MHC complexes is important in understanding the immune response to SARS-CoV-2 vaccinations and the viral immune evasion strategies employed by VOCs (Variants of concern). Mutations in T cell epitopes can disrupt recognition by TCRs, which highlights the importance of modeling and studying the spatial structures of TCR – peptide MHC complexes.

There are several main factors that guide antigen recognition in TCR-peptide-MHC complex: placement of the peptide in the MHC groove, pairing of TCR- alpha and beta chains, specific orientation of pMHC complex against T-cell receptor and selective interaction of TCR residues with MHC and peptide.

Most studies suggest that TCRs recognize peptide-MHC complexes via CDR1-3 loops. While CDR1 and CDR2 loops stabilize binding with MHC molecules and target small parts of a protein, most of the interaction between TCR and antigen occurs via CDR3, which are omega-loops of 10-20 amino acids (28, 29). Also, it has been shown that CDR3 loop sequence’s mid-region creates most of the contacts with peptide due to the loop and peptide geometry. Additionally, both alpha and beta chains contribute to antigen recognition, although sometimes this contribution is disproportionate (30).

The present study reports the results of large-scale template-based modeling of the VDJdb, which is a curated database of TCR sequences with known specificities. We aim at traversing the set of TCR:pMHC specificity records that differ by a fixed number of amino acid substitutions from the original template, as it was shown that 1-3 consequent substitutions rarely impact antigen specificity (31). Our primary aim is to establish a pipeline that can be easily employed to create accurate and reliable spatial structures for studies that report massive sequencing of TCRs specific to a panel of antigens. By applying this pipeline to the existing database we aim at providing access to structural data for immunologists and biologists unfamiliar with *in silico* structural modeling. We also demonstrate, as a proof-of-concept, that such modeling can be used to study features of residue interactions in TCR:pMHC complexes, and report statistics of various physicochemical interactions between TCR and peptide.

## 2 Methods

### 2.1 Identification of CDR3 sequences clusters recognizing the same epitope

Clustering of CDR3 sequences from the T-cell receptor sequences that recognize the same epitope according to the VDJdb database (6) was performed in three major steps. First, a “core” of a cluster was designated by one of the CDR3 sequences presented in a PDB structure. Second, new CDR3 sequences (neighbors) were placed in the cluster if they could be generated step-by-step introducing single amino acid substitutions one at a time. The CDR3 sequences should have the same lengths, so no deletions or insertions were allowed. Additionally, the V- and J- genes of corresponding TCRs should be the same as the one carrying the “core” CDR3 loop. Pairing of TCRα - TCRβ chains was not taken into account. Third, the Hamming distance between the core and each of the neighbor CDR3 sequences in the cluster was limited to 3. Thus, the mutation path from the CDR3 sequence from the PDB database and the most diverse sequence in the cluster should consist of not more than 3 consequent single mutations. The validation of our proposed modeling approach and the explanation of why we selected a limit of 3 mutations is discussed in **Supplementary Note 1**.

### 2.2 Step-by-step modeling of CDR3 loops

A Rosetta release-215 package (32) was used to implement an *in silico* single amino acid mutation modeling approach. The Rosettascripts mover “MutateResidue” was used to replace selected residues while retaining main chain heavy atom positions according to the initial template structures, modifying only side chain atoms.

Further structural and energy optimization was performed using the Minimization or PackRotamers movers implemented in RosettaScripts. The energy minimization procedure has the potential to cause significant changes and “overfitting” in the spatial organization of the entire complexes or their contacting parts. To address this issue, the minimization protocol incorporated C-alpha atoms coordinate constraints with a coord_dev parameter set to 0.5. The L-BFGS minimization algorithm was utilized with the *ref2015* score function. Additionally, the repacking protocol of Rosettascripts was employed to optimize contacting residues in modeled TCR-peptide-MHC complexes. Repacking was performed for CDR3 loop and peptide residues situated closer than 10Å from each other, as these residues were considered as contacting in modeled complexes.

### 2.3 Structural data analysis

Identification of close CDR3 and peptide residues in modeled complexes was performed using in house Java and Bash scripts. Contacting parts of residues (side and main chains) were identified using Residue Interaction Network Generator approach (RING) (33).

The energy values for contacting CDR3 - peptide interfaces in both the initial templates and the modeled structures were calculated using the Rosetta interface_energy application. The calculation also included the determination of per-residue impacts. In this process, the residues of the CDR3 loop of interest were designated as “-face1”, while the peptide residues were designated as “-face2”. Energy values were calculated using three variations of Rosetta ref2015 scoring function: “full” ref2015 energy function with all default energy terms included, and two “limited” variations, set by lists of selected terms. The first preset (“Large patch”) included *fa_atr* (attractive portion of the Lennard Jones potential), *fa_sol* (Lazaridis-Karplus solvation energy), *hbond_sr_bb* (h-bond energy, short-range backbone-backbone), *hbond_lr_bb* (bond energy, long-range backbone-backbone), *hbond_bb_sc* (h-bond energy, backbone-sidechain) and *hbond_sc* (h-bond energy, sidechain-sidechain) energy terms. The second (“Small patch”) consisted of the same terms as the “Large patch”, except for *hbond_sr_bb* and *hbond_bb_sc*.

Amino acid descriptors and indices were calculated using “Peptides” (BLOSUM similarity matrix indices, Kidera factors, VHSE, Cruciani properties, zScales, FASGAI) and “HDMD” (Atchley factors) packages in R (34, 35).

### 2.4 Statistical analysis and machine learning

Statistical analysis of computed energy values of the modeled structures and properties of contacting amino acid residues in TCR-peptide-MHC complexes was performed using R packages. RFE analysis was performed on the training set consisting of 94 amino acid descriptors, calculated for contacting amino acid residues in CDR3 loops of TCRs and peptides to select the most important descriptors for predicting the target variable. Random forest algorithm was then used to train a model, which was used to make predictions on the testing set. Per-residue energy values were grouped into 5 clusters and used as target variables during the RFE analysis and RF modeling. The performance of the model was evaluated using confusion matrix analysis. Calculations and data preparation were performed using Caret and RandomForest packages in R.

## 3 Results

### 3.1 Building a compendium of TCR: pMHC models for VDJdb

**Figure 1:**
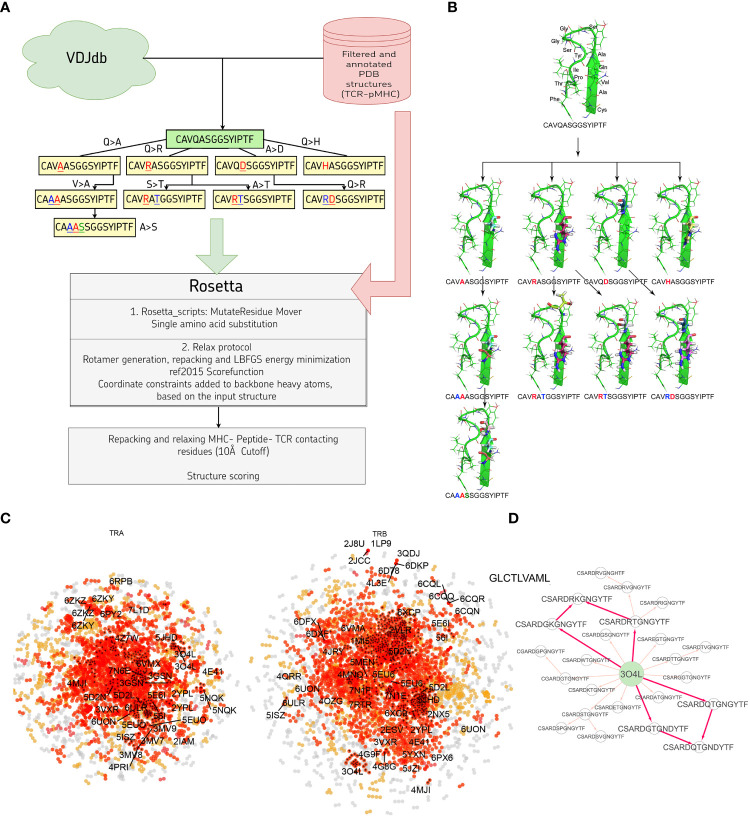
**(A)** Pipeline used in the stepwise amino acid mutation modeling approach applied in the present study. **(B)** Mutation path in one of the studied CDR3 clusters starting from a known 2NX5 PDB structure (36) carrying CAVQASGGSYIPTF CDR3α loop. **(C)** CDR3 sequence similarity map of VDJdb, layout of a graph with edges connecting CDR3 sequences that differ by no more than a single amino acid mismatch. CDR3 sequences having PDB templates are shown with labels. Orange points show VDJdb entries that were classified as belonging to a sequence homology motif (described in (37)), red points are connected components built around PDB structure templates, TCR:pMHC records connected by no more than 3 subsequent amino acid substitutions to a PDB that were modeled in present study are shown with black crosses. **(D)** An example of the mutation pathways in which the same CDR3 loops could be modeled using different intermediate sequences. The corresponding pathways are shown with bold red arrows.

In this study we developed a bioinformatic pipeline for modeling T cell receptor CDR3 loops by stepwise introduction of single amino acid mutations to known spatial structures of complexes used it to construct 1585 TCR-peptide-MHC models and analyzed contacting residues between CDR3 loops and peptides in modeled complexes. The pipeline applied in our approach is shown in **Figures 1A, B**. In the first step we curated a pre-processed and annotated (see Methods) database of TCR-peptide-MHC structures from PDB and built clusters of annotated CDR3 sequences recognizing the same epitope from VDJdb, using CDR3 loops from known PDBs as cores. In the next stage we calculated pathways for consequent introduction of single amino acid substitutions to cover all sequences in selected clusters, starting from known PDB structures. The actual extent of VDJdb coverage by our modeling approach is depicted in **Figure 1C**, and the modeling paths are presented in **Supplementary Figure S1**.

Using 1030 unique mutation pathways, all initial structures were altered and further optimized using Rosetta protocols. As a result, we built 1585 models based on 29 template structures from the PDB database, which contain 247 unique sequences of CDR3 loops for TCR-alpha and 485 for TCR-beta chains. The total number of modeled structures is greater than the number of unique pathways or unique CDR3 sequences because the same CDR3 loops carrying, for example, two or three substitutions can be modeled through various intermediate sequences (**Figure 1D**). Additionally, different initial PDB templates could be used to model the same CDR3 loops. All modeled CDR3 sequences are presented in **Supplementary Figure S2**, and the number of models according to TCR chains, presence of contact with peptide, and type of contact are presented in **Supplementary Table S1**.

The initial templates are presented in **Table 1**. It is worth noting that some PDB templates carried identical TCR chains. During further analysis, models built based on such templates were grouped, and their calculated spatial and energy properties were averaged. These grouped structures would be referred to as “non-redundant”.

**Table 1:** Details for initial PDB templates and the number VDJdb entries modeled as structures.

Template annotation							Modeled structures	
PDB	Peptide	TCRα V-gene	TCRα J-gene	TCRβ V-gene	TCRβ J-gene	MHC gene	Unique CDR3α	Unique CDR3β
5nqk	ELAGIGILTV	TRAV12-2*01	TRAJ45*01	TRBV19*01	TRBJ2-2*01	HLA-A2	1	–
5nht	ELAGIGILTV	TRAV12-2*01	TRAJ45*01	TRBV19*01	TRBJ2-2*01	HLA-A2	4	–
2nx5	EPLPQGQLTAY	TRAV1-2*01	TRAJ6*01	TRBV10-3*01	TRBJ1-5*01	HLA-B35	5	4
1mi5	FLRGRAYGL	TRAV26-2*01	TRAJ52*01	TRBV7-8*01	TRBJ2-7*01	HLA-B8	–	5
5isz	GILGFVFTL	TRAV24*01	TRAJ27*01	TRBV19*01	TRBJ2-7*01	HLA-A2	–	2
5euo	GILGFVFTL	TRAV27*01	TRAJ37*01	TRBV19*01	TRBJ2-7*01	HLA-A2	61	216
2vlr	GILGFVFTL	TRAV27*01	TRAJ42*01	TRBV19*01	TRBJ2-7*01	HLA-A2	29	79
1oga	GILGFVFTL	TRAV27*01	TRAJ42*01	TRBV19*01	TRBJ2-7*01	HLA-A2		
2vlj	GILGFVFTL	TRAV27*01	TRAJ42*01	TRBV19*01	TRBJ2-7*01	HLA-A2		
2vlk	GILGFVFTL	TRAV27*01	TRAJ42*01	TRBV19*01	TRBJ2-7*01	HLA-A2		
5e6i	GILGFVFTL	TRAV35*01	TRAJ37*01	TRBV27*01	TRBJ2-2*01	HLA-A2	5	2
5jhd	GILGFVFTL	TRAV38-2/DV8*01	TRAJ52*01	TRBV19*01	TRBJ1-2*01	HLA-A2	1	83
3o4l	GLCTLVAML	TRAV5*01	TRAJ31*01	TRBV20-1*01	TRBJ1-2*01	HLA-A2	17	23
3mv7	HPVGEADYFEY	TRAV20*01	TRAJ58*01	TRBV9*01	TRBJ2-2*01	HLA-B35	7	6
3mv8	HPVGEADYFEY	TRAV20*01	TRAJ58*01	TRBV9*01	TRBJ2-2*01	HLA-B35		
3mv9	HPVGEADYFEY	TRAV20*01	TRAJ58*01	TRBV9*01	TRBJ2-2*01	HLA-B35		
4pri	HPVGEADYFEY	TRAV20*01	TRAJ58*01	TRBV9*01	TRBJ2-2*01	HLA-B35		
2ypl	KAFSPEVIPMF	TRAV5*01	TRAJ13*01	TRBV19*01	TRBJ1-2*01	HLA-B57	3	18
4g9f	KRWIIMGLNK	TRAV14DV4*01	TRAJ21*01	TRBV6-5*01	TRBJ1-1*01	HLA-B27	–	2
1ao7	LLFGYPVYV	TRAV12-2*01	TRAJ24*01	TRBV6-5*01	TRBJ2-7*01	HLA-A2	1	–
4ftv	LLFGYPVYV	TRAV12-2*01	TRAJ24*01	TRBV6-5*01	TRBJ2-7*01	HLA-A2		
3gsn	NLVPMVATV	TRAV24*01	TRAJ49*01	TRBV6-5*01	TRBJ1-2*01	HLA-A2	3	–
5d2l	NLVPMVATV	TRAV24*01	TRAJ49*01	TRBV7-2*01	TRBJ2-5*01	HLA-A2	8	20
5d2n	NLVPMVATV	TRAV26-2*01	TRAJ43*01	TRBV7-6*01	TRBJ1-4*01	HLA-A2	17	19
6vmx	RPPIFIRRL	TRAV24*01	TRAJ37*01	TRBV4-1*01	TRBJ1-2*01	HLA-B7	2	–
4mji	TAFTIPSI	TRAV17*01	TRAJ22*01	TRBV7-3*01	TRBJ2-2*01	HLA-B51	1	1
7n6e	YLQPRTFLL	TRAV12-1*01	TRAJ43*01	TRBV19*01	TRBJ2-2*01	HLA-A2	68	–
7n1f	YLQPRTFLL	TRAV12-2*01	TRAJ30*01	TRBV7-9*01	TRBJ2-7*01	HLA-A2	15	34
7rtr	YLQPRTFLL	TRAV12-2*01	TRAJ30*01	TRBV7-9*01	TRBJ2-7*01	HLA-A2		

Dash (“-”) indicates there were no VDJdb records with 1-3 mismatches we could model using our approach.

### 3.2 Analysis of CDR3: peptide contacts in modeled structures

Using a 5Å cutoff distance, contacting (or “interacting”) residues of “mutated” CDR3 loops and peptides were identified in all modeled structures and structures obtained from the PDB database. It was shown that only about half of them carried substitution in contacting positions: approximately 48% (763 models out of 1585) in all TCR-peptide-MHC complex models and ~51% (551 models out of 1085) in the non-redundant set of structures.

Since all the modeled TCRs in the cluster have the same specificity as TCR in the core crystal structure, we expect that modeled amino acid substitutions should not dramatically influence the binding between TCR and epitope. That might be the reason why many amino acid variations were found in non-contacting positions - they are simply distal and do not alter TCR-pMHC interface. Additionally, it can be assumed that the remaining substitutions in contacting positions should not dramatically change the affinity level of binding between CDR3 loops and epitopes in the way that can alter their specificity. We assume that as we consider only single amino acid substitutions between CDR3 sequences belonging to the cluster of highly similar TCRs recognizing the same epitope.

CDR3–peptide interactions were analyzed in all initial templates. For each structure the most energetically valuable positions in CDR3 loops were identified through per-residue energy calculations for CDR3 alpha- and beta-peptide interfaces. Positions in CDR3 loops with the lowest energy values were selected. Similar to other energy calculations described in the article, three types of energy scoring functions were used: “full” Rosetta interface energy and two of its limited variations: “Large patch” and “Small patch” presets. When these scorings were ambiguous in terms of minimal energy value, we included all candidate positions (**Table S2**). These results were compared with estimated frequencies of amino acid variations in each position on CDR3 loops in our modeled structures. It was found out that only 11% of all mutations and 23% of mutations in contacting residues were in energetically valuable positions. In the non-redundant set the rate was slightly higher: 15% and 29% respectively. These results also indicate that substitutions mainly occur in regions of CDR3 loops that are not very important for epitope recognition, thus preserving specificity and affinity of binding in TCR-peptide-MHC complexes.

All amino acid pairs involved in CDR3-peptide interactions were identified and counted in all modeled and 152 original structures of human TCR-peptide-MHCI complexes. Interactions between amino acid residues of CDR3 loops and peptides were classified based on their distances and contacting parts: main chain or side chain groups. We considered either all contacts (a single TCR residue can contact several peptide residues located in distance<5Å) or only closest contacting residues (for each TCR residue only a single contact with the closest residue in the peptide was considered). Four groups were formed based on these parameters (1): all contacting residue pairs, (2) closest contacting residues (for each TCR residue only a single contact with the closest residue in the peptide was considered), (3) residues contacting through side chain groups, and (4) closest contacting residues through side chain groups. For the third and fourth groups we considered only contacts in which at least one of the residues interact through a side chain group. Analysis of side chain groups is crucial because practically all physicochemical features of amino acid residues are “encoded” in side chain groups.

The number of all found contacting amino acid pairs between CDR3 loops and peptides in original and modeled structures is presented in **Figure S3**.

It can be seen that, even though there are some new contacting residue pairs in the modeled structures that were not found in the original structures, the majority was not unique.

We visually inspected the contacting residues in the CDR3 alpha and beta clusters modeled from the core sequences presented in 7RTR and 7N1F original PDB structures. These structures contain TRAV12-2*01/TRAJ30*01 TCRα chain with “CAVNRDDKIIF” CDR3, TRBV7-9*01/TRBJ2-7*01 TCRβ chain with “CASSPDIEQYF” CDR3, and YLQPRTFLL SARS-CoV2 epitope presented by MHCI. Using selected clusters, 54 unique single amino acid substitutions were modeled: 16 substitutions were introduced to the CDR3 loop of TCR alpha and 38 to TCR beta.

Analyzing all these modeled substitutions, it was found out that in most cases they were in non-contacting positions according to the corresponding original structures. Contacting residues were only changed to similar amino acids, while more dissimilar ones only appeared in positions where contact with the peptide was formed by main chain atoms. Therefore, changes of side chain groups should not influence CDR3-peptide binding.

**Figure 2:**
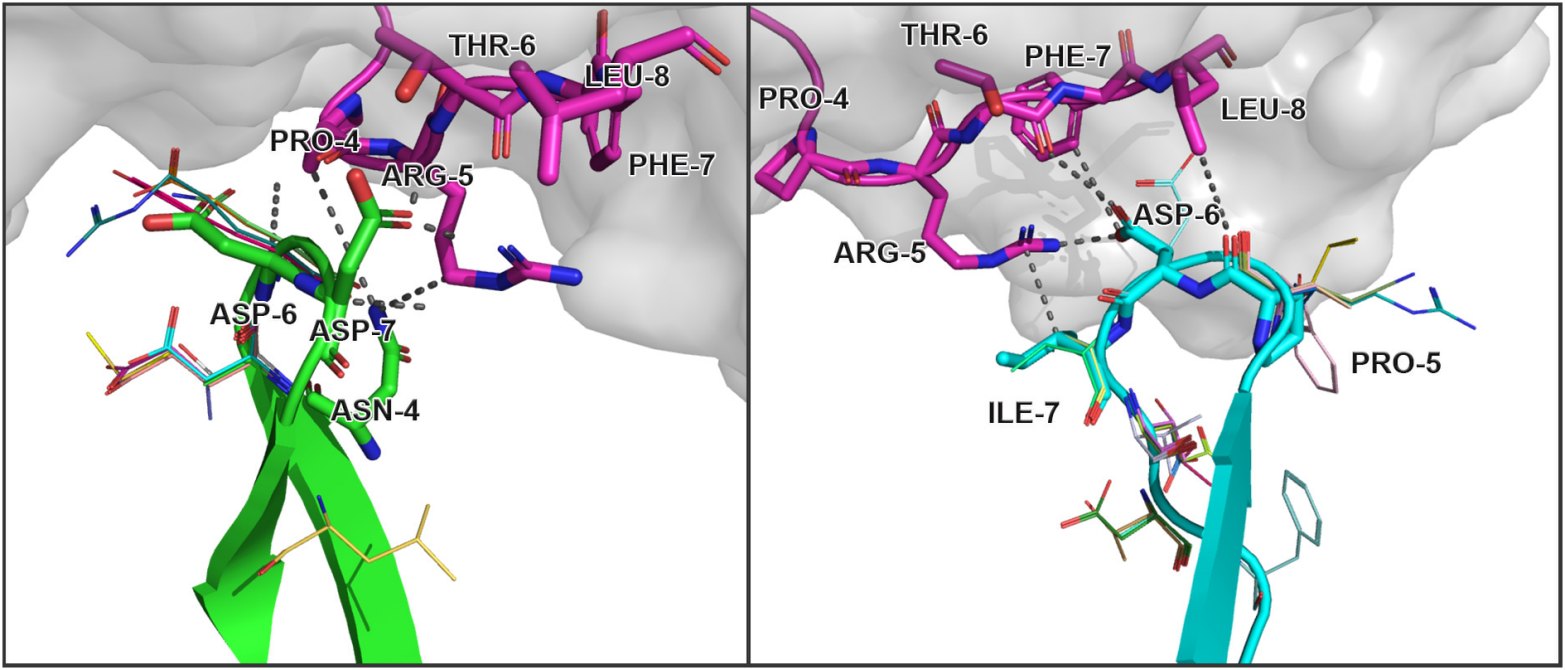
Modeled amino acid substitutions in CDR3 loops in TCR:peptide:MHC complexes containing SARS-CoV-2 spike epitopes YLQPRTFLL. *Contacting residues are represented as sticks, modeled substitutions are represented as lines and close contacts are illustrated as dashed sticks. CDR3 loop of TCRα is colored green, TCRβ is colored cyan, and the peptide residues are colored magenta*.

The modeled substitutions are shown in **Figure 2**, and corresponding information about the contacting residues between the CDR3 loops and peptide is presented in **Table 2**.

**Table 2:** Contacting residues in modeled TCR:peptide:MHC complexes, built based on 7RTR and 7N1F original PDB structures.

CDR3			Peptide		Interaction			Modeled substitutions
chain	residue	position	residue	position	Type of interaction	cdr3	peptide	
TCRα	Asn	4	Pro	4	VDW	side chain	side chain	none
			Arg	5	VDW	side chain	side chain	
	Asp	6	Pro	4	VDW	main chain	main chain	multiple
			Arg	5	VDW	main chain	side chain	
	Asp	7	Pro	4	VDW	side chain	main chain	none
			Arg	5	HBOND	side chain	side chain	
			Thr	6	HBOND	side chain	side chain	
TCRβ	Pro	5	Leu	8	VDW	main chain	side chain	multiple
	Asp	6	Arg	5	IONIC	side chain	side chain	GLU
			Thr	6	VDW	side chain	main chain	
			Phe	7	VDW	side chain	side chain	
	Ile	7	Arg	5	VDW	side chain	side chain	VAL, SER

Annotation of interactions is presented in accordance with the results of the analysis of residue interaction networks. Provided amino acid numbering starts from the beginning of the corresponding substructure – CDR3 loop or peptide.

During the inspection of the selected modeled structures (**Table 2**) it was observed that only two contacting positions carried multiple variations of amino acids: Asp-6 in the CDR3α and Pro-5 in the CDR3β loop. In both cases, the side chain groups of the mutated residues were oriented towards the MHC molecule instead of the epitope. Two other positions carried one or two variants of substituted amino acids that were quite similar to the original in terms of their physicochemical properties: Glu instead of Asp in the 6th position and Val or Ser instead of ILE in the 7th position of CDR3β loop.

### 3.3 Correlation of CDR3: peptide binding energy values and amino acid properties

It was previously shown (38) that the most important energy terms during the calculation of free binding energies between TCRs and peptides were the attractive van der Waals impact energy, solvation energy, and side-chain – side-chain hydrogen bond energy, while the repulsive van der Waals term was insignificant. Thus, in our research, energy values for contacting residues and interfaces between modified CDR3 loops and peptides in all modeled complexes were calculated using full Rosetta scoring functions and two of its limited variations: “Large patch” and “Small patch” presets (see the “Methods” section). As all models were built using stepwise introduction of single amino acid substitutions, we calculated the changes in the values of interface energies between complexes that differ by 1 amino acid. These changes in energy values represent the impacts of point amino acid variations on the affinity of complexes. Henceforth that type of values would be referred to as “dEnergy”.

#### 3.3.1 Correlation between BLOSUM scores and dEnergy values

Two types of BLOSUM matrices (BLOSUM62 and BLOSUM100) were used to study the consistency between amino acid substitution scores and their impact on dEnergy in modeled complexes. Pearson’s correlations were calculated between absolute values of free energy adjustments caused by mutation and one of three modified BLOSUM indices of corresponding substitutions: Clustered Target Frequencies (QIJ), Clustered Scoring Matrix in Bit Units (SIJ) or most commonly used Clustered Scoring Matrix in 1/2 Bit Units (BLA). Adjusted index values were calculated as follows:


$$\frac{BLOS_{i}\left(substitution\right)-BLOS_{i}\left(from\right)-BLOS_{i}\left(to\right)}{2}$$

where *BLOS_i_* (substitution) - BLOSUM index of studied substitution

*BLOS_i_* (from) - BLOSUM index of a match of origin residue

*BLOS_i_* (to) - BLOSUM index of a match of new residue

As BLOSUM scores represent the ratio of the likelihood of two amino acids being exchanged with biological significance, it is more reasonable to compare them with absolute values of dEnergy without taking into consideration its sign. Thus, we can evaluate the impact of substitutions to similar or dissimilar amino acids. Energy values were calculated using the full Rosetta scoring function and “Large patch” and “Small patch” presets.

Correlations were analyzed for all mutated residues in CDR3 loops and separately for substitutions in only contacting positions. Top 20 corresponding correlation values and p-values are presented in **Table 3**.

**Table 3:** Correlation values, calculated for BLOSUM scores and absolute values of dEnergy after the introduction of amino acid substitutions in modeled complexes.

Blosum index	All mutations		Contacting mutations		Energy scoring	Optimization
	Pearson correlation	P-value	Pearson correlation	P-value		
BLA.62.v2	-0.136	**2.03E-04**	-0.219	**7.76E-06**	Small patch	Minimized
BLA.62.v2	-0.135	**2.36E-04**	-0.205	**2.69E-05**	Large patch	Minimized
SIJ.62.v2	-0.112	**0.002**	-0.182	**2.16E-04**	Small patch	Minimized
SIJ.100.v2	-0.097	**0.008**	-0.165	**6.67E-11**	Small patch	Minimized
SIJ.62.v2	-0.107	**0.004**	-0.164	**0.001**	Large patch	Minimized
BLA.100.v2	-0.096	**0.009**	-0.164	**1.86E-10**	Small patch	Minimized
BLA.62.v2	-0.135	**4.34E-05**	-0.161	**3.07E-04**	Large patch	Repacked
SIJ.100.v2	-0.084	**0.022**	-0.137	**0.005**	Large patch	Minimized
BLA.100.v2	-0.083	**0.024**	-0.136	**0.006**	Large patch	Minimized
BLA.62.v2	-0.123	**1.79E-04**	-0.133	**0.003**	Small patch	Repacked
BLA.62.v2	-0.055	0.119	-0.128	**0.008**	Rosetta	Minimized
SIJ.62.v2	-0.117	**4.01E-04**	-0.126	**0.005**	Large patch	Repacked
SIJ.62.v2	-0.050	0.154	-0.113	**0.019**	Rosetta	Minimized
SIJ.100.v2	-0.107	**0.001**	-0.110	**0.014**	Large patch	Repacked
SIJ.62.v2	-0.082	**0.011**	-0.109	**0.015**	Rosetta	Repacked
SIJ.100.v2	-0.085	**0.009**	-0.102	**0.023**	Rosetta	Repacked
BLA.100.v2	-0.106	**0.001**	-0.099	**0.026**	Large patch	Repacked
BLA.100.v2	-0.038	0.287	-0.099	**0.040**	Rosetta	Minimized
BLA.62.v2	-0.071	**0.028**	-0.097	**0.029**	Rosetta	Repacked
SIJ.100.v2	-0.040	0.261	-0.097	**0.044**	Rosetta	Minimized

Statistically significant test results (P ≤ 0.05) are highlighted in bold font.

**Figure 3:**
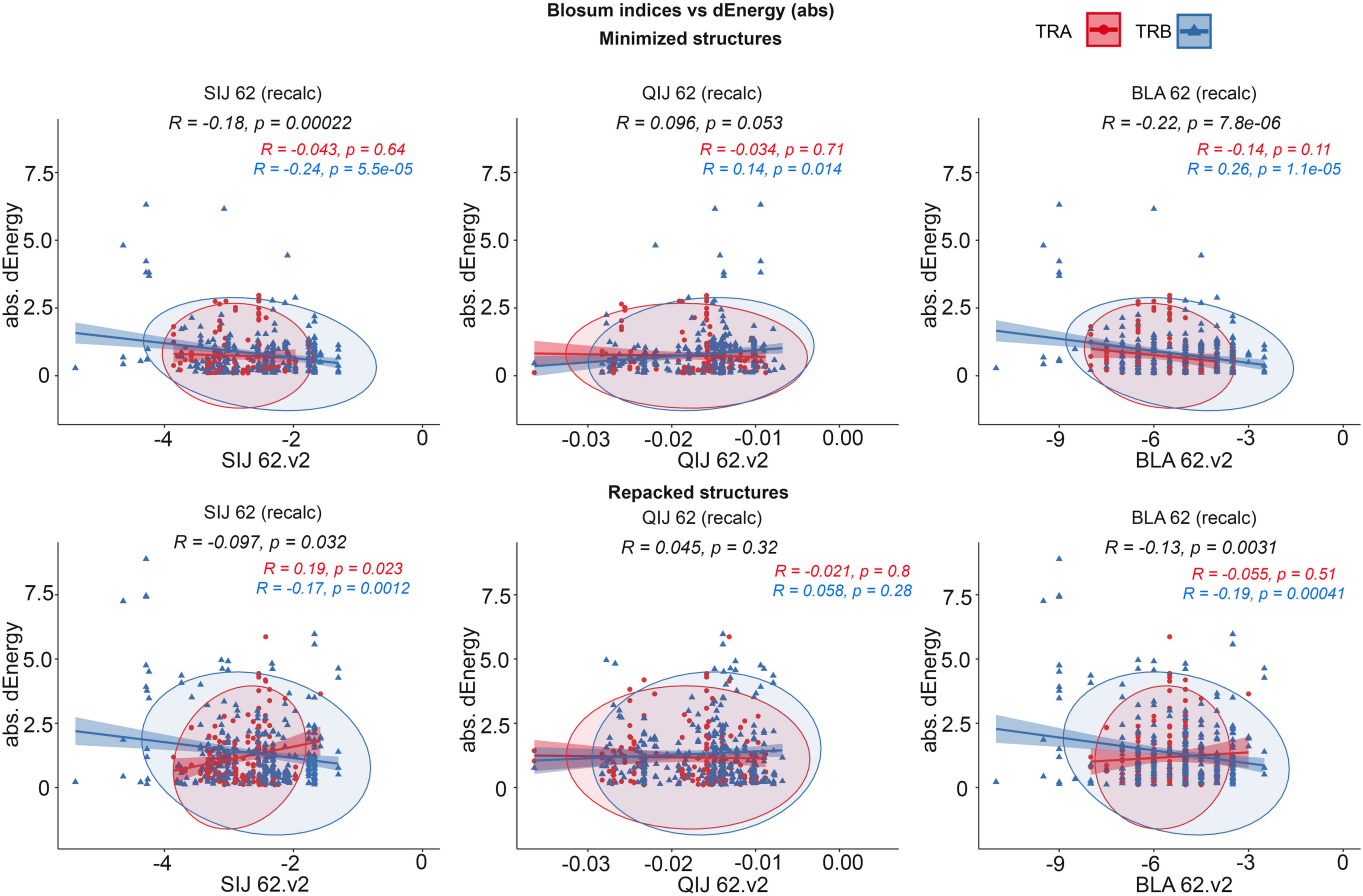
Correlation of BLOSUM62 matrix indices and absolute values of dEnergy, calculated for modeled structures. *Corresponding values for TCRα and TCRβ are colored red and blue*.

It was observed that the correlations between different BLOSUM62 or BLOSUM100 indexes and absolute delta energy values were very poor overall. The best correlation was observed between BLA.62 values and the absolute value of dEnergy, calculated using the “Small patch” preset in structures that carried mutations in contacting positions. Pearson correlation values (R coefficient) was -0.22 and -0.13 for minimized and repacked structures respectively. The results are presented in **Figure 3**. It can also be observed that correlation is better for TCRβ chains, which corresponds to previously shown results that beta-chains are more crucial for the specificity of peptide recognition and binding.

These results show that standard BLOSUM matrices might not be as informative in the analysis of full CDR3 sequences, their clusterization and prediction of binding with specific peptides. The highest correlation was observed when only containing residues were considered. This fact highlights that instead of aligning and analyzing full CDR3 sequences, special attention should be paid to contacting parts.

Despite the low correlations overall, it was mentioned that using energy minimization as the final optimization of models led to a slight improvement of corresponding values compared to repacking. This can be explained by the “fitting” of contacting interfaces in TCR-peptide-MHC complexes after repacking of “mutated” models, which resulted in “smoothing” (reduction) of the substitution impact.

#### 3.3.2 Influence of remoteness of contacting amino acids on dEnergy

We additionally analyzed the impact of the distance between substituted residues and peptides on dEnergy of TCR-peptide-MHC binding. In cases when mutated residue in CDR3 interacted with more than one residue of epitope, we used minimal distance values. It was shown that the total Pearson correlation values between distance and absolute values of dEnergy in minimized modeled structures were -0.27, -0.39 and -0.41 for energy values calculated using the full Rosetta scoring function, “Large-” and “Small patch” presets respectively. The negativity in correlation values indicates that the more distant a modeled substitution is from the peptide, the less it impacts dEnergy values, which makes biophysical sense.

**Figure 4:**
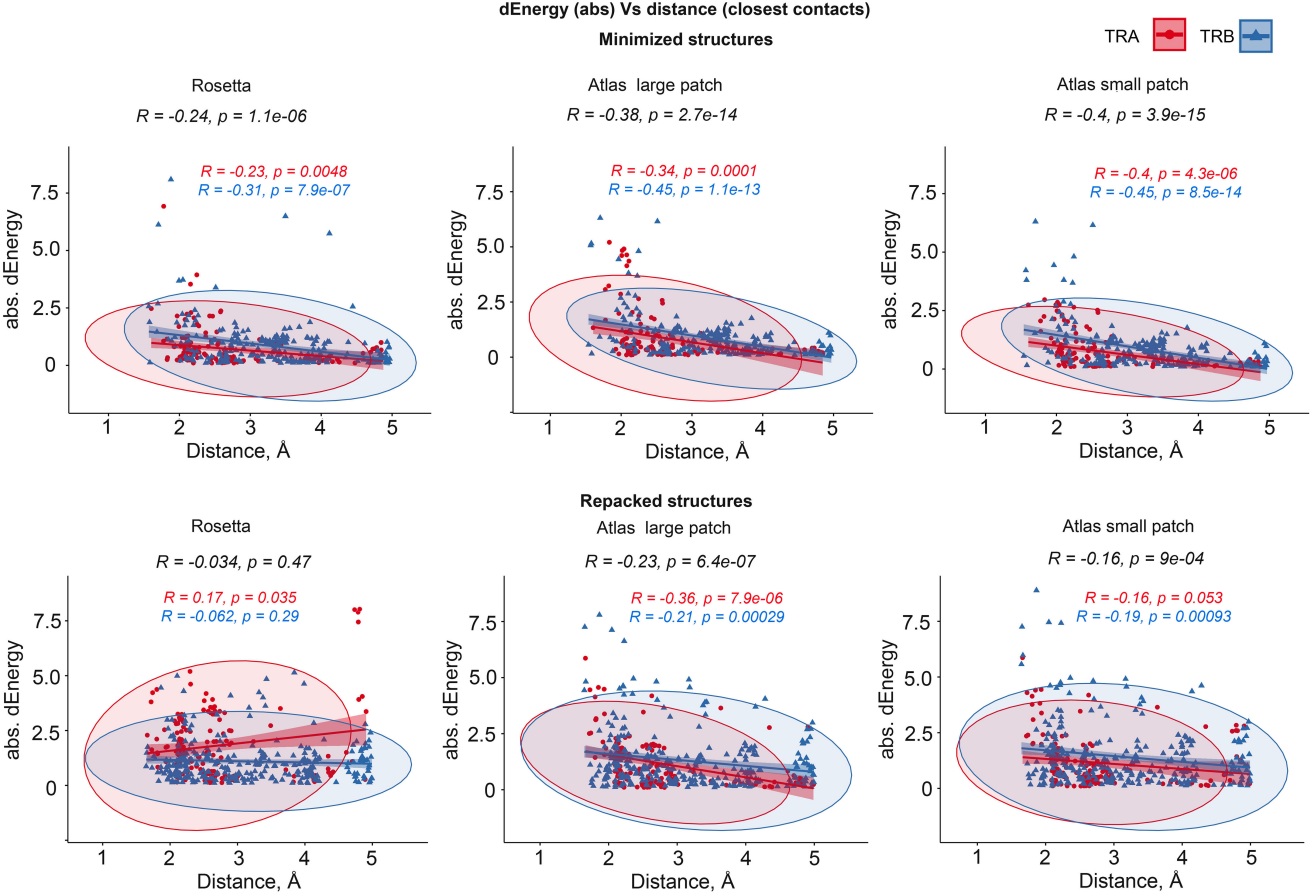
Correlation between the remoteness of contacting residues in CDR3-peptide interface and absolute dEnergy values. Overall correlation values are presented in black, and linear fittings calculated for TCR-alpha and TCR-beta chains independently are presented in red and blue respectively.

Analyzing these dependencies for TCRα and TCRβ separately, it can be seen that correlation was better for TCRβ in comparison with TCRα. These results indicate that CDR3 loops of beta-chains might have a greater impact on peptide recognition than TCR-alpha’s. Corresponding correlations, calculated for repacked structures were smaller: -0.074, -0.26 and -0.18. This decrease in correlation also supports the concept of “fitting” of mutated CDR3 residues after repacking optimization. Correlation graphs are presented in **Figure 4**.

#### 3.3.3 Correlation between physicochemical properties of cognate CDR3 and epitope residues

Using “Peptides” and “HDMD” R packages, seven groups of amino acid descriptors were calculated for the contacting residues in CDR3 loops and peptides in original and mutated TCR-peptide-MHC complex structures. It should be mentioned that only mutated positions were taken into account. The descriptor groups were as follows: BLOSUM indices (BLOS1-BLOS10), Kidera factors (*KF1-KF13*), VHSE (*VHSE1- VHSE 8*), Cruciani properties (*PP1-PP3*), zScales (*Z1-Z5*), FASGAI (*F1-F6*) and Atchley factors (PAH, *PSS*, MS, CC, EC) (39).

The majority of calculated indices can be grouped according to the specific physicochemical features they represent: hydrophobicity, steric properties, electronic, and secondary structure features (**Table 4**).

**Table 4:** Groups of studied amino acid descriptors.

Property	Amino acid indices
Hydrophobicity	Z1, PP2, F1, VHSE1, VHSE2, Blos1, KF4, KF10
Steric properties	a.ms, Z2, F3, VHSE3, VHSE4, Blos1, Blos2, Blos3, KF2
Electronic properties	a.pah, a.ec, Z3, PP1, F6, VHSE5, VHSE6, VHSE7, VHSE8
Secondary structure	a.pss, F2, Blos1, Blos3, KF1, KF3, KF5, KF8

Names of the Atchley indices were shortened using “a.” prefix.

The rest of the descriptors, for example, BLOS4 and BLOS9 correlate with several properties and the specific one cannot be prioritized.

**Figure 5:**
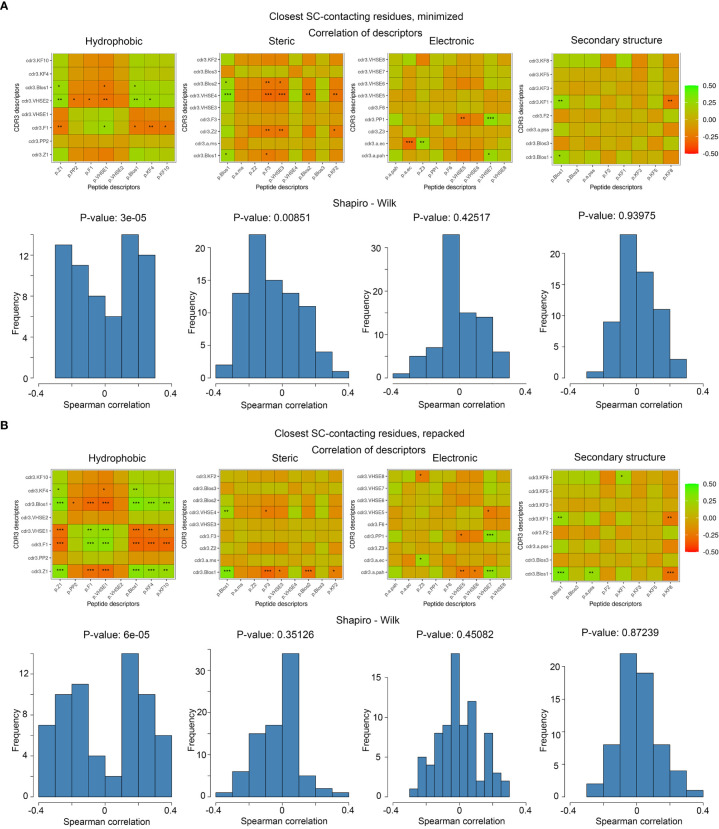
Correlations of calculated descriptors between contacting residues of CDR3 loops and epitopes. The presented correlation analysis was performed for the closest contacting residues, interacting through at least one side-chain group in **(A)** minimized and **(B)** repacked models. Paired correlation values are represented as waffle plots, and their distribution in groups is shown as histograms. Statistical significance of correlation is labeled with “*”, “**” and “***” for P-values< 0.05, 0.01 and 0.001 respectively.

To analyze the consistency between properties of contacting residues in CDR3 loops and epitopes, we performed rank correlation analysis of the corresponding factors and indices. Spearman correlations were calculated within selected groups of descriptors for all interacting residues, the closest contacting residues, and amino acid pairs interacting through at least one side chain group. The most valuable correlations in groups of descriptors were identified using the Shapiro-Wilk test. Paired correlations and their distribution for selected groups of indices are presented in **Figure 5**, and best correlations are listed in **Table 5**.

**Table 5:** Top correlation values between calculated CDR3 and epitope residues indices.

Descriptor type	CDR3 descriptor	Peptide descriptor	Correlation	p-value	p-value (adjusted)	Optimization and contact type
A. Positive correlations						
Steric	VHSE4	p.Blos1	0.32	1.09E-09	4.15E-06	Minimized, closest, side-chain
Hydrophobic	Blos1	Z1	0.33	4.93E-11	1.86E-07	Repacked, closest, side-chain
	Z1	Blos1	0.31	6.34E-10	2.40E-06	
	F1	VHSE1	0.31	9.46E-10	3.57E-06	
	Blos1	KF4	0.30	3.88E-09	1.46E-05	
B. Negative correlations						
Steric	VHSE6	VHSE5	-0.30	1.13E-08	4.27E-05	Minimized, closest, side-chain
	VHSE6	F5	-0.31	4.09E-09	1.54E-05	
	Blos1	F3	-0.31	8.2E-10	3.09E-06	Repacked, closest, side-chain
Hydrophobic	Blos3	F3	-0.30	2.95E-09	1.11E-05	Repacked, closest, side-chain
	Z1	VHSE1	-0.31	1.96E-09	7.41E-06	
	F1	Z1	-0.31	6.1E-10	2.30E-06	
	F1	KF4	-0.32	5.59E-10	2.11E-06	
	F1	Blos1	-0.32	3.22E-10	1.21E-06	
	F1	KF10	-0.32	2.82E-10	1,07E-06	
	Blos1	VHSE1	-0.33	8.12E-11	3.07E-07	
Electronic	a.ec	a.ec	-0.31	6.59E-09	2.49E-05	Minimized, closest, side-chain

The best correlation values were calculated for the hydrophobic and steric groups of descriptors in minimized structures, and their distribution was non-normal, according to the Shapiro–Wilk test (p-values were 3x10^-5^ and 0.0085, respectively). After repacking of modeled structures, which led to structural fitting of contacting residues and displacement of their side-chain groups, the correlations of steric descriptors became weaker, while the correlations of hydrophobic descriptors still remained highly significant (p-value = 6x10^-5^).

### 3.4 Identifying residue binding features in CDR3: peptide interface using machine learning

At the final stage we performed RFE (Recursive Feature Elimination) analysis and applied the Random Forest (RF) algorithm to make predictive models for the per-residue contacting energy values for amino acid residue pairs in CDR3 loops and peptides. RF was selected as it can handle high-dimensional data and is unlikely to do overfitting, while also allowing to easily assess feature importance which is needed in exploratory analysis.

To include a wider range of valid pairwise contacts between CDR3 loops and TCRs, we calculated amino acid descriptors and energy values for known TCR-peptide-MHC complexes available in the PDB database. In total, we added 152 structures of human T-cell receptors with peptides and MHC-I triple complexes to the analysis.

As our modeled structures were optimized using energy minimization or repacking of contacting residues, corresponding structures were treated separately and combined with selected resolved structures from the PDB database. The two extended datasets, containing 94 amino acid descriptors and affinity values (per-residue energies) for all the studied contacting residues were divided into training and testing sets in 70% to 30% proportion. Affinity values were converted to 5 groups (factors) using quantile values.

Discretizing the target variables is a common step in machine learning procedures such as Random Forest, and there are several reasons for doing so. First, dividing the affinity values into meaningful groups can make it easier to interpret the results. Second, discretizing the values can help mitigate the effects of outliers and noise in the data. Finally, it can be useful for classification tasks, such as predicting whether a given interaction is “strong” or “weak”.

During RFE analysis of the extended datasets containing either minimized or repacked modeled structures, we identified 10 and 13 variables, respectively, as the most important predictors of the affinity values. These descriptors are presented in **Table 6**.

**Table 6:** Selected amino acid descriptors during Random Feature Elimination procedure.

Minimized + original	Repacked + original
**p.F1 (H)**	**p.F1 (H)**
**cdr3.VHSE1 (H)**	**cdr3.VHSE1 (H)**
**cdr3.Z1 (H)**	**cdr3.Z1 (H)**
cdr3.KF10 (H)	**cdr3.KF10 (H)**
**cdr3.KF4 (H)**	cdr3.KF4 (H)
cdr3.VHSE7 (E)	cdr3.VHSE7 (E)
cdr3.Blos8	cdr3.VHSE8 (E)
**cdr3.a.ms (S)**	cdr3.a.ms (S)
p.a.cc	cdr3.F2 (SS)
cdr3.KF7	**cdr3.KF7**
	cdr3.Blos7
	cdr3.F5
	cdr3.Blos10

The top,5 descriptors according to the calculated importance are highlighted in bold font. The properties represented by the descriptors are labeled as follows: H, hydrophobic; E, electronic; S, steric; SS, secondary structure.

Upon analyzing the selected descriptors, it is noticeable that most of them belonged to amino acids from CDR3 loops, and the majority of the top 5 descriptors were related to the hydrophobic properties of the studied residues.

Evaluating the models built for the two selected datasets using testing data showed that the quality of their predictions was relatively similar for repacked and minimized structures.

The accuracy of the model built using minimized structures was 0.4212 with a 95% confidence interval of 0.3905 - 0.4524, and the accuracy of the model using repacked structures was 0.448 with a 95% confidence interval of 0.417 - 0.4793. The Kappa statistic for the “minimized” model was 0.2765, and for the “repacked” model, it was 0.3098. The slightly higher accuracy of the model built using the extended data set of repacked structures and the higher Kappa statistic indicates better agreement between predicted and actual values compared to the second model built using minimized structures. The McNemar’s Test P-Value was also lower for the “repacked” model, suggesting that it may be a more reliable model.

## 4 Discussion

In this study we proposed an *in silico* approach for template-based modeling of highly similar CDR3 loops in full TCR-peptide–MHC complexes using stepwise introduction of single amino acid substitutions. Using our approach, we modeled 1585 structures based on 29 templates and studied the non-redundant set of structures by grouping identical TCR alpha and beta chains and utilized the dataset to study the determinants of antigen recognition on a single-residue level. Our results greatly extend the number of available structures with different hypervariable CDR3 sequence variants, including 16 synthetic TCR alpha structures and 38 TCR beta structures modeled based on 7RTR and 7N1F SARS-CoV-2 antigen-TCR complex templates.

Upon analyzing these structures, we observed that widely used standard BLOSUM62 and BLOSUM100 scores have little correlation with the impact of amino acid mutations in CDR3 loops on TCR–epitope binding energy as long as those substitutions appear in non-contacting parts of loops. This finding suggests that the analysis and comparison of CDR3 sequences in context of epitope recognition should mainly consider contacting residues, and conventional full-length sequence alignment alone is not sufficient to compare binding affinity of CDR3 sequences to the same antigen.

Also, we found that contacting residues in CDR3b can have a greater effect on TCR–peptide recognition than those in CDR3a loops. This conclusion is based on the analysis of the impact of CDR3 amino acid variations on interaction energy values, depending on their remoteness from peptides and correlation between BLOSUM indices and calculated absolute values of dEnergy of interacting interfaces. In both cases corresponding properties and values of CDR3b residues showed better correlations in comparison to CDR3a.

Analysis of physicochemical properties of contacting residues in CDR3 loops and epitopes, described by different descriptors, showed that hydrophobicity may be an important factor governing recognition and affinity of binding between CDR3 loops and epitopes in TCR-peptide-MHC complexes, yet in all analysis correlation values were moderate. This is in agreement with the fact that all studied amino acid residue substitutions have limited impact on binding of CDR3 loops to peptides in order to preserve T-cell receptor’s recognition.

The pipeline described in this paper can be reused when more templates and/or TCR specificity data becomes available, greatly extending the amount of available structural data on antigen recognition by TCRs. Possible extensions to this work include models in which the antigen is substituted by a similar peptide that binds the same MHC in order to monitor for potential TCR cross-reactivity and perform in-depth study of the determinants of TCR specificity using molecular dynamics.

## Data availability statement

The datasets presented in this study can be found in online repositories. The names of the repository/repositories and accession number(s) can be found below: https://zenodo.org/record/8143087, 7845844.

## Author contributions

VDJdb data preparation, molecular modeling and data analysis: DS, MS, VK. Manuscript draft and editing: DS, MS, VK, IZ, DC. Supervised the study: MS, IZ, DC. All authors contributed to the article and approved the submitted version.
