# Supplementary material for: Large-scale template-based structural modeling of T-cell receptors with known antigen specificity reveals complementarity features
Source: Front Immunol. 2023 Aug 15;14:1224969. doi: 10.3389/fimmu.2023.1224969 (PMC10464843; doi:10.3389/fimmu.2023.1224969)
Supplement: Supplementary file 1 [file DataSheet_1.pdf]

Supplementary Figures and Tables

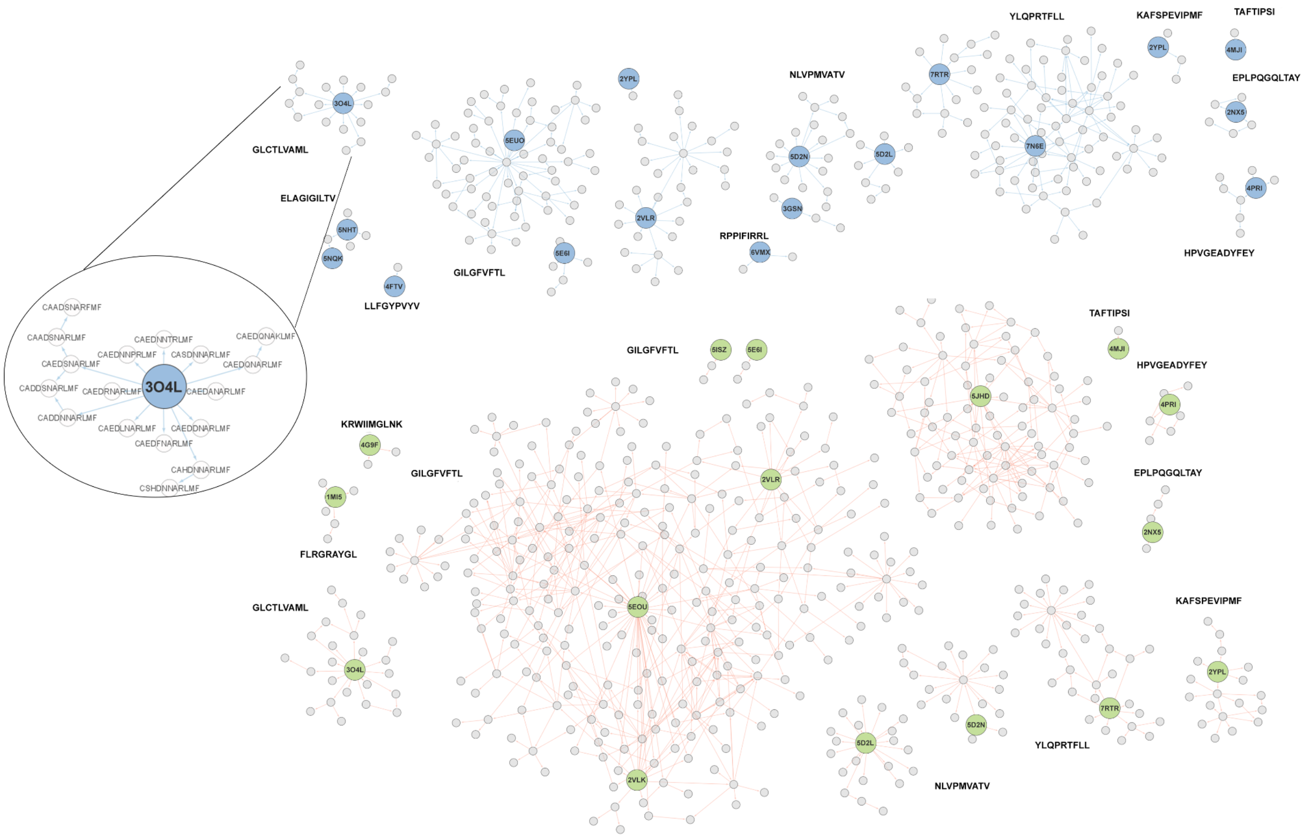

Figure S1 Graph with paths used for stepwise modeling of CDR3 loops. *CDR3α sequences are connected with blue edges and sequences of CDR3β – red. Initial templates are presented as circles with PDB identifiers and are colored blue in CDR3α and green in CDR3β clusters. Epitope labels are placed near corresponding connected components.*

CDR3α	CDR3β	Peptide	PDBs
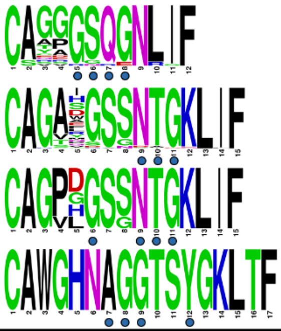	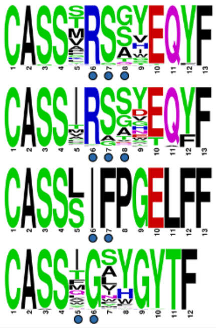	GILGFVFTL	2vlj 2vlk 2vlr 1oga<br>5euo<br>5e6i<br>5jhd
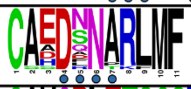	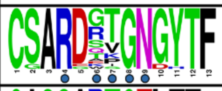	GLCTLVAML	3o4l
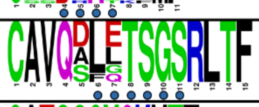	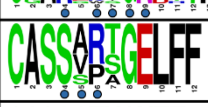	HPVGEADYFEY	3mv7 3mv8 3mv9 4pri
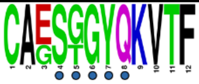	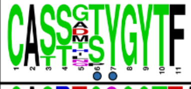	KAFSPEVIPMF	2ypl
	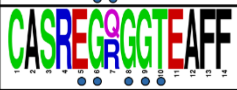	KRWIIMGLNK	4g9f
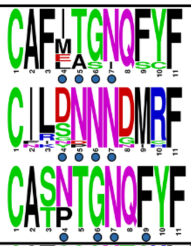	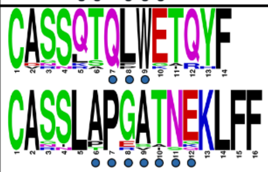	NLVPMVATV	5d2l<br>5d2n<br>3gsn
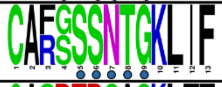		RPPIFIRRL	6vmx
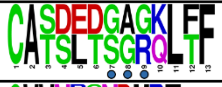		TAFTIPSI	4mji
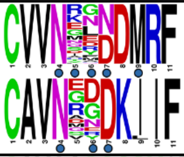	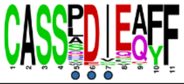	YLQPRTFLL	7n6e<br>7n1f 7rtr
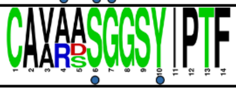	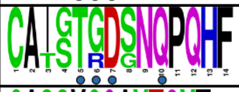	EPLPQGQLTAY	2nx5
	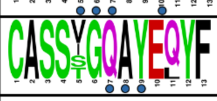	FLRGRAYGL	1mi5
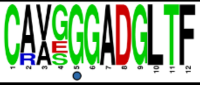		ELAGIGILTV	5nht

Figure S2. Logo-formatted sequences of CDR3 loops in modeled structures. *Residues, contacting with peptides are marked with blue circles.*

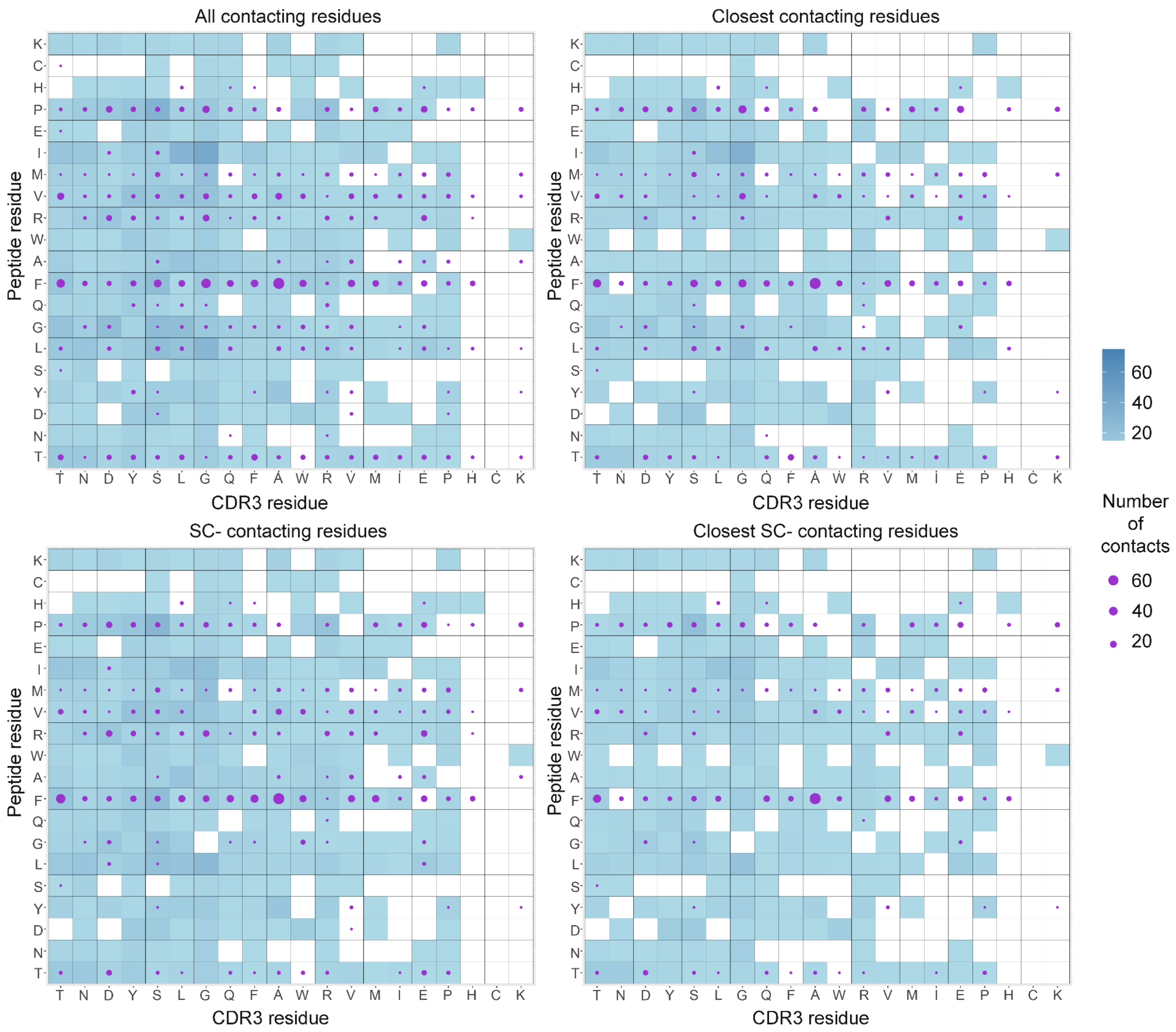

Figure S3. Contacting amino acid pairs identified in original and modeled structures. *Contacts in the original structures are represented as squares, while contacts in the modeled structures are represented as circles. The number of identified contacting pairs is illustrated as a gradient fill for the original structures, and the size of circles represents the results for the modeled structures. The results for all contacting residue pairs are presented in the upper-left corner; closest contacting residue pairs are shown in the upper right corner. Amino acid residues, contacted by at least one side chain group are in the down-left corner, and the results for closest amino acid residues contacting by at least one side chain group are presented in the down-right corner.*

Table S1. Number of paired CDR3 – epitope contacting residues in modeled structures.

"All contacts"- all contacting pairs formed by mutated residue of CDR3 loops with epitope amino acids, situated closer than 5Å apart.

"SC- contacting"- contacting pairs formed by mutated residue of CDR3 loops with epitope amino acids, situated closer than 5Å apart. At least one residue in the pair interacts by its side chain group.

"Closest contacts" – only the closest residue of epitope selected for each mutated residue of CDR3 loop in each modeled structure.

"Closest SC-contacts" - only the closest, interacting by at least one side chain group, residues of epitope selected for each mutated residue of CDR3 loop in each modeled structure.

	**All contacts**		**SC-contacts**		**Closest contacts**		**Closest SC-contacts**	
Minimized	**1022**		**584**		**537**		**346**	
	CDR3a	CDR3b	CDR3a	CDR3b	CDR3a	CDR3b	CDR3a	CDR3b
	302	720	203	381	176	361	114	232
Repacked	**1060**		**635**		**569**		**370**	
	CDR3a	CDR3b	CDR3a	CDR3b	CDR3a	CDR3b	CDR3a	CDR3b
	323	737	195	440	176	393	101	269

Table S2. Most energetically valuable contacting amino acid residues in the original PDB structures.

The residues were selected based on the minimum of per-residue calculated interface energy values. As far as the calculations were performed using several variations of scoring functions, some PDB structures carry more than one most valuable amino acid pairs for the specific CDR3 loop.

**PDB**	**TCR Chain**	**CDR3 sequence**	**CDR3 position**	**CDR3 residue**	**Peptide position**	**Peptide residue**	**peptide**
1ao7	TRA	CAVTTDSWGKLQF	6	D	4	G	LLFGYPVYV
1ao7	TRA	CAVTTDSWGKLQF	7	S	5	Y	LLFGYPVYV
1ao7	TRB	CASRPGLAGGRPEQYF	7	L	8	Y	LLFGYPVYV

1mi5	TRA	CILPLAGGTSYGKLTF	5	L	6	A	FLRGRAYGL
1mi5	TRB	CASSLGQAYEQYF	8	A	7	Y	FLRGRAYGL
1oga	TRA	CAGAGSQGNLIF	7	Q	4	G	GILGFVFTL
1oga	TRB	CASSSRSSYEQYF	6	R	7	F	GILGFVFTL
2nx5	TRA	CAVQASGGSYIPTF	10	Y	4	P	EPLPQGQLT AY
2nx5	TRB	CATGTGDSNQPQHF	5	T	8	L	EPLPQGQLT AY
2vlj	TRA	CAGAGSQGNLIF	7	Q	4	G	GILGFVFTL
2vlj	TRB	CASSSRSSYEQYF	7	S	5	F	GILGFVFTL
2vlj	TRB	CASSSRSSYEQYF	6	R	7	F	GILGFVFTL
2vlk	TRA	CAGAGSQGNLIF	7	Q	4	G	GILGFVFTL
2vlk	TRB	CASSSRSSYEQYF	6	R	7	F	GILGFVFTL
2vlr	TRA	CAGAGSQGNLIF	7	Q	4	G	GILGFVFTL
2vlr	TRB	CASSSRASYEQYF	6	R	7	F	GILGFVFTL
2ypl	TRA	CAVSGGYQKVTF	7	Y	8	I	KAFSPEVIP MF
2ypl	TRA	CAVSGGYQKVTF	8	Q	6	E	KAFSPEVIP MF
2ypl	TRB	CASTGSYGYTF	7	Y	7	V	KAFSPEVIP MF
3gsn	TRA	CARNTGNQFYF	4	N	5	M	NLVPMVATV
3gsn	TRB	CASSPVTGGIYGYTF	8	G	5	M	NLVPMVATV
3gsn	TRB	CASSPVTGGIYGYTF	7	T	8	T	NLVPMVATV
3mv7	TRA	CAVQDLGTSGSRLTF	10	G	7	D	HPVGEADYF EY
3mv7	TRA	CAVQDLGTSGSRLTF	10	G	6	A	HPVGEADYF EY
3mv7	TRB	CASSARSGELFF	6	R	8	Y	HPVGEADYF EY

3mv8	TRA	CAVQDLGTSGSRLTF	10	G	6	A	HPVGEADYFEY
3mv8	TRA	CAVQDLGTSGSRLTF	6	L	3	V	HPVGEADYFEY
3mv8	TRB	CASSARSGELFF	6	R	8	Y	HPVGEADYFEY
3mv9	TRA	CAVQDLGTSGSRLTF	10	G	6	A	HPVGEADYFEY
3mv9	TRA	CAVQDLGTSGSRLTF	6	L	3	V	HPVGEADYFEY
3mv9	TRB	CASSARSGELFF	6	R	8	Y	HPVGEADYFEY
3o4l	TRA	CAEDNNARLMF	6	N	4	T	GLCTLVAML
3o4l	TRA	CAEDNNARLMF	7	A	4	T	GLCTLVAML
3o4l	TRB	CSARDGTGNGYTF	4	R	6	V	GLCTLVAML
3pqy	TRA	CILSGGSNYKLTF	9	Y	7	R	SSLENFRAYV
3pqy	TRB	CASSFGREQYF	6	G	8	A	SSLENFRAYV
4ftv	TRA	CAVTTDSWGKLQF	6	D	5	Y	LLFGYPVYV
4ftv	TRA	CAVTTDSWGKLQF	6	D	4	G	LLFGYPVYV
4ftv	TRB	CASRPGLMSAQPEQYF	7	L	8	Y	LLFGYPVYV
4g9f	TRA	CAMRDLRDNFNKFYF	10	F	6	M	KRWIIMGLNK
4g9f	TRB	CASREGLGGTEAFF	10	T	6	M	KRWIIMGLNK
4mji	TRA	CATDDDSARQLTF	9	R	6	P	TAFTIPSI
4mji	TRA	CATDDDSARQLTF	9	R	4	T	TAFTIPSI
4mji	TRB	CASSLTGGGELFF	6	T	6	P	TAFTIPSI
4pri	TRA	CAVQDLGTSGSRLTF	6	L	3	V	HPVGEADYFEY
4pri	TRB	CASSARSGELFF	6	R	8	Y	HPVGEADYF

							EY
5d2l	TRA	CAFITGNQFYF	4	I	5	M	NLVPMVATV
5d2l	TRA	CAFITGNQFYF	5	T	4	P	NLVPMVATV
5d2l	TRB	CASSQTQLWETQYF	9	W	7	A	NLVPMVATV
5d2l	TRB	CASSQTQLWETQYF	7	Q	7	A	NLVPMVATV
5d2n	TRA	CILDNNNDMRF	6	N	5	M	NLVPMVATV
5d2n	TRB	CASSLAPGTTNEKLFF	9	T	5	M	NLVPMVATV
5d2n	TRB	CASSLAPGTTNEKLFF	8	G	5	M	NLVPMVATV
5e6i	TRA	CAGPGGSSNTGKLIF	10	T	4	G	GILGFVFTL
5e6i	TRB	CASSLIYPGELFF	6	I	7	F	GILGFVFTL
5e6i	TRB	CASSLIYPGELFF	7	Y	5	F	GILGFVFTL
5euo	TRA	CAGAIGPSNTGKLIF	9	N	4	G	GILGFVFTL
5euo	TRA	CAGAIGPSNTGKLIF	10	T	5	F	GILGFVFTL
5euo	TRB	CASSIRSSYEQYF	6	R	7	F	GILGFVFTL
5isz	TRA	CAFDTNAGKSTF	7	A	4	G	GILGFVFTL
5isz	TRA	CAFDTNAGKSTF	6	N	4	G	GILGFVFTL
5isz	TRB	CASSIFGQREQYF	8	Q	5	F	GILGFVFTL
5jhd	TRA	CAWGVNAGGTSYGKLT F	12	Y	5	F	GILGFVFTL
5jhd	TRB	CASSIGVYGYTF	5	I	5	F	GILGFVFTL
5nht	TRA	CAVGGGADGLTF	5	G	4	G	ELAGIGILTV
5nht	TRB	CASSQGLAGAGELFF	7	L	7	I	ELAGIGILTV
5nht	TRB	CASSQGLAGAGELFF	10	A	5	I	ELAGIGILTV
5nqk	TRA	CAGGGGADGLTF	5	G	4	G	ELAGIGILTV
5nqk	TRB	CASSQGLAGAGELFF	5	Q	8	L	ELAGIGILTV
5nqk	TRB	CASSQGLAGAGELFF	7	L	7	I	ELAGIGILTV

5wlg	TRA	CATVYAQGLTF	7	Q	4	L	SQLLNAKYL
5wlg	TRB	CASSDWGDTGQLYF	6	W	6	A	SQLLNAKYL
5wlg	TRB	CASSDWGDTGQLYF	5	D	7	K	SQLLNAKYL
6vmx	TRA	CAFGSSNTGKLIF	6	S	4	I	RPPIFIRRL
6vmx	TRB	CASSQDLFTGGYTF	9	T	5	F	RPPIFIRRL
6vmx	TRB	CASSQDLFTGGYTF	6	D	8	R	RPPIFIRRL
6vmx	TRB	CASSQDLFTGGYTF	9	T	6	I	RPPIFIRRL
7n1f	TRA	CAVNRDDKIIF	7	D	5	R	YLQPRTFLL
7n1f	TRA	CAVNRDDKIIF	4	N	5	R	YLQPRTFLL
7n1f	TRB	CASSPDIEQYF	7	I	5	R	YLQPRTFLL
7n1f	TRB	CASSPDIEQYF	6	D	5	R	YLQPRTFLL
7n6e	TRA	CVVNRNNDMRF	6	N	4	P	YLQPRTFLL
7n6e	TRB	CAGQVTNTGELFF	6	T	5	R	YLQPRTFLL
7n6e	TRB	CAGQVTNTGELFF	7	N	7	F	YLQPRTFLL
7rtr	TRA	CAVNRDDKIIF	7	D	5	R	YLQPRTFLL
7rtr	TRA	CAVNRDDKIIF	4	N	5	R	YLQPRTFLL
7rtr	TRB	CASSPDIEQYF	7	I	5	R	YLQPRTFLL
7rtr	TRB	CASSPDIEQYF	6	D	5	R	YLQPRTFLL
