# Supplementary material for: Large-scale template-based structural modeling of T-cell receptors with known antigen specificity reveals complementarity features
Source: Front Immunol. 2023 Aug 15;14:1224969. doi: 10.3389/fimmu.2023.1224969 (PMC10464843; doi:10.3389/fimmu.2023.1224969)
Supplement: Supplementary file 2 [file DataSheet_2.pdf]

Supplementary Note 1

Validation of the proposed stepwise single amino acid substituting modeling approach

Materials and methods

TCR-pMHC homology modeling.

Modeling of the selected TCR structures was performed using our approach, TCR-pMHC modeling *(1)* service, TCRModel *(2)* algorithm implemented in the Rosetta package and TCRModel2 *(3)*. The models were selected based on their sequence similarity to known templates from the PDB database. CDR3 loops were modeled by introducing less than 6 amino acid substitutions starting from the known template, and the resulting “target” TCR chain should also be resolved in the PDB database. During modeling using TCRModel, target structures were eliminated from the templates library. TCR-pMHC modeling was performed using the online service with default settings (http://www.cbs.dtu.dk/services/TCRpMHCmodels/index.php). These models were used to validate our modeling and optimization approaches.

In addition to energy minimization and repacking of contacting residues, loop refinement was performed for our models using a kinematic protocol implemented in the LoopRemodel Rosettascripts mover. During the refinement procedure the four terminal residues (two residues at the N-terminus and two - at C-terminus) of the CDR3 loop were fixed, while the rest of the backbone and side chains were allowed to be flexible.

Molecular dynamics

Molecular dynamics simulations were carried out using Gromacs-2020.5 software package (4). The systems were neutralized by adding Na^+ and Cl^- , and solvated with TIP3P water models. MD calculations were performed using the Amber99-SB force field. Energy minimization was sequentially performed in vacuum and in solvation for 150,000 steps. Subsequently, temperature was increased to 300K and pressure was set to 1 atm using NVT and NPT ensembles, respectively. Molecular dynamics simulations were performed on 50 ns trajectories with 2 fs time steps.

Results

Minimization vs. repacking of TCR-pMHC contacting area.

Initial visual inspection of some previously modeled complexes using our approach revealed sterical issues that can occur during spatial positioning of large amino acid residues instead of small ones, and their orientation was altered depending on the final optimization step. For example, finalizing repacking optimization led to the misplacement of Arg and Tyr side chain groups in mutated β-chain CDR3 loop from 2VLR PDB structure (**Figure SN1**) compared to a structure received after energy minimization only. To validate the stability of these modeled structures, we performed molecular dynamics calculations for three selected pMHC-TCR complexes: two modeled structures with the “CASSIRWAYEQYF” CDR3 loop of the beta-chain of TCR, built based on 2VLR PDB structures with just energy minimization or repacking of contacting residues as the final optimization step; and the original 2VLR PDB structure that was used as an initial template for the modeling process.

During the analysis of the MD trajectories, the most attention was paid to the displacement of peptide-MHC complex with respect to TCR since the observed displacement of side chain groups in contacting residues might influence the stability of the TCR-pMHC interaction. It was shown that in the original TCR-pMHC complex structure (PDB id: 2VLR) the pMHC position was the most stable (**Figure SN1D**). RMSD values for the peptide-MHC molecules were in the range of 3.5-5A during molecular dynamics.

On the other hand, the modeled complex that was built using only energy minimization as the final optimization step was unstable (**Figure SN1D**), and the MHC molecule with the peptide dramatically shifted from their initial position during MD (**Figure SN1B**). Such low affinity of this modeled complex might be explained by a bad structural “complementary” in the TCR-pMHC contacting interface due to the unfavorable positioning of replaced side chain groups.

Changes in RMSD values for the MHC-peptide molecules in the third (repacked) complex showed that at the beginning of molecular dynamics, the pMHC complex shifted from its initial pose and returned back after 15 - 20 ns of MD.The alteration of MHC-TCR docking pose is shown in **Figure SN1C**.

The primary aim of employing molecular dynamics was to demonstrate the impact of distinct optimization methods, specifically repacking and minimization, on the stability of TCR-peptide-MHC complexes. Our results clearly indicate that, even within the relatively short timescale of 50 ns, there was noticeable variation in stability between complexes optimized using different procedures. However, it should be noted that while the observed stability within this concise timescale was evident, we cannot definitively determine whether the "repacked" complex will remain stable on larger trajectories.

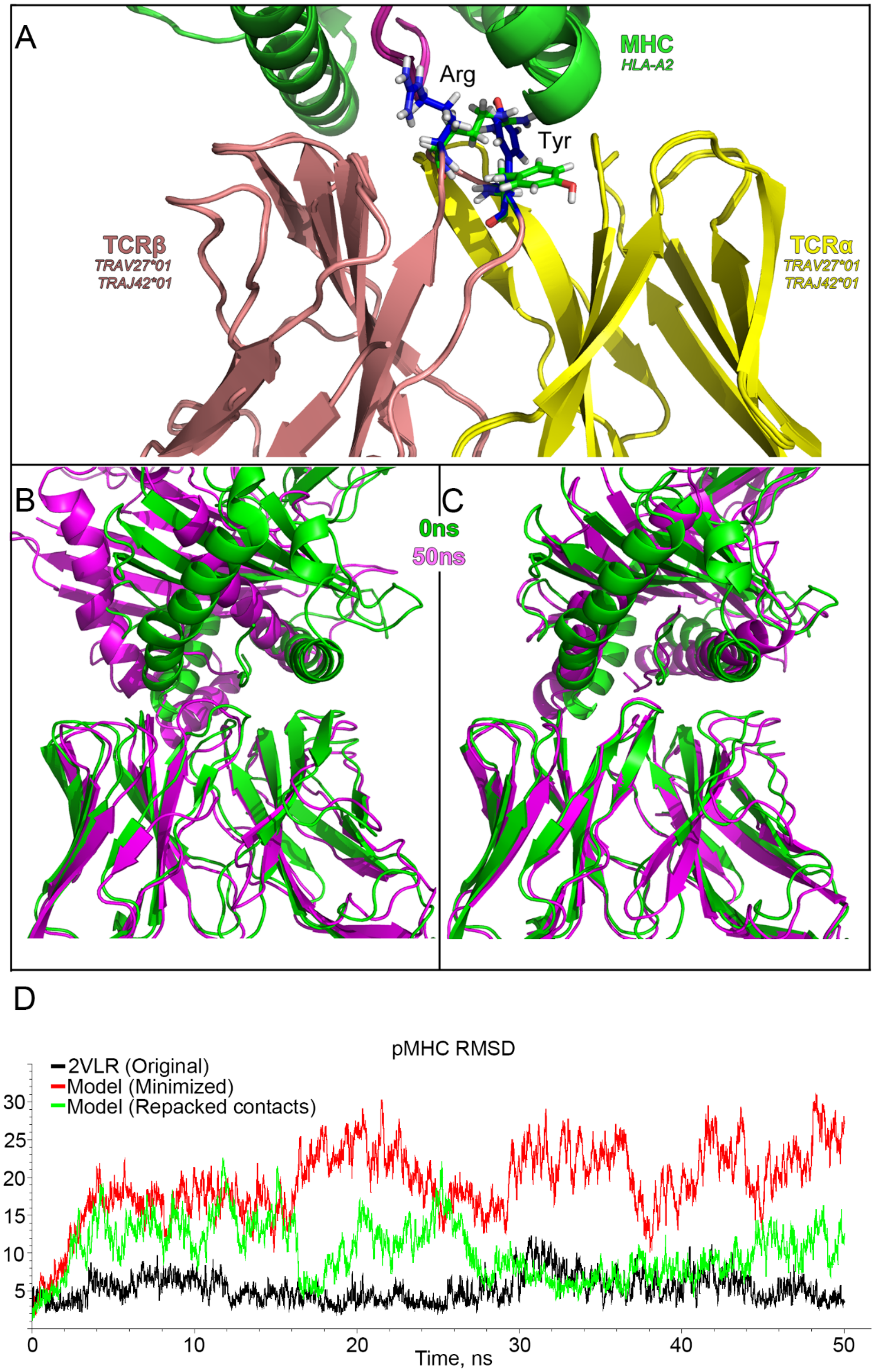

A
MHC
HLA-A2
Arg
Tyr
TCRβ
TRAV27*01
TRAJ42*01
TCRα
TRAV27*01
TRAJ42*01
B
C
0ns
50ns
D
pMHC RMSD
2VLR (Original)
Model (Minimized)
Model (Repacked contacts)
RMSD, Å
Time, ns

E

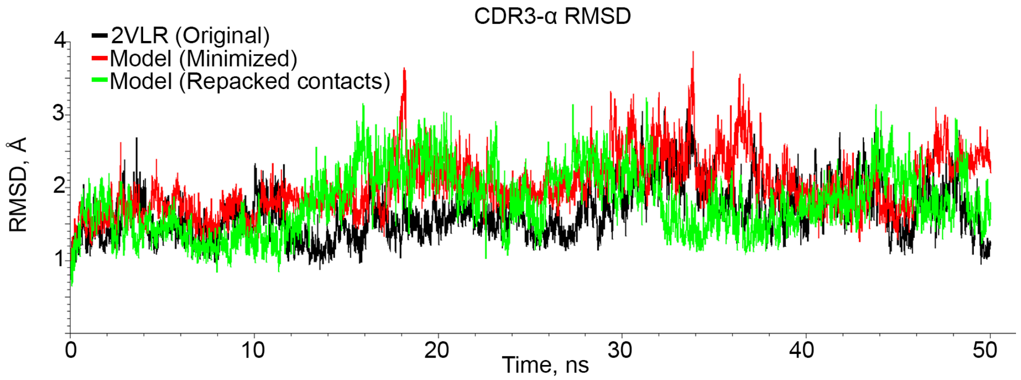

F

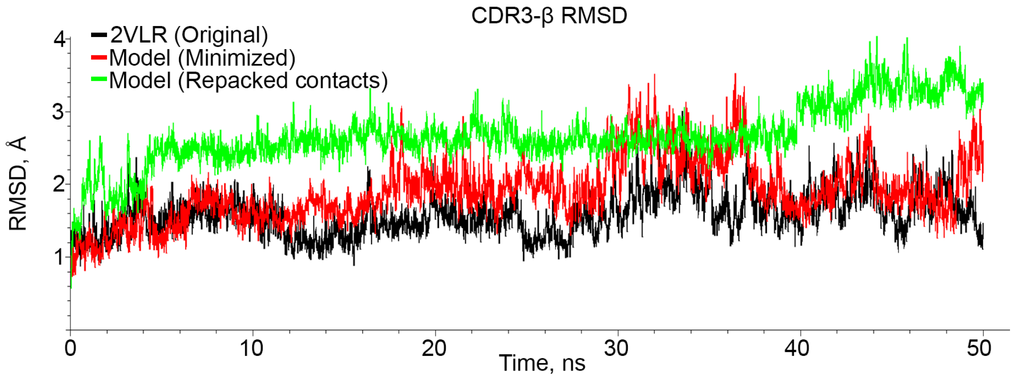

Figure SN1 *Stability of the original and modeled TCR-pMHC complexes and structural comparison of CDR3 loops after energy minimization or repacking of contacting residues.*

A) Spatial displacement of Arg and Tyr side chain groups after repacking of contacting residues in pMHC – TCR complex built from 2VLR*. Repacked amino acids are colored blue, mutated and just minimized structures are green.*

B) Displacement of the peptide-MHC complex during molecular dynamics of just minimized modeled structures and C) after repacking of the contacting interface.

D) RMSD values, calculated for peptide-MHC complexes, CDR3a loops (F) and CDR3b loops (E) during molecular dynamics. Values of the original PDB structure (2VLR) are represented as a black lines, minimized modeled structure - as red, and repacked modeled structure - as green. All structures of complexes were aligned by TCR chains.

The analysis of the RMSD values for CDR3 loops in the original 2VLR PDB structure revealed that the CDR3-alpha and CDR3-beta loops exhibited significant stability, with RMSD values of approximately 1-1.5A. In contrast, the loops in the structure, optimized by energy minimization, displayed somewhat greater conformational flexibility. RMSD values for the CDR3-alpha and CDR3-beta loops fluctuated within 1.5 – 2.5A, occasionally peaking at

3-3.5A. A similar pattern was observed for the CDR3-alpha loop in the repacked structure, with a notable decrease in RMSD values after 30ns of MD, occasionally reaching as low as 1A. The CDR3-beta loop in the repacked mutated structure demonstrated stability, maintaining RMSD values around 2.5-3A following a small initial shift.

However, given the length of these loops, the observed deviations ranging from 1.5 to 3.5A do not represent significant shifts in their backbone conformation.

The results of our molecular dynamics analysis suggest that repacking of contacting residues might be a necessary step while modeling CDR3 loops in TCR-pMHC complexes using our step-by-step single mutation approach. This might assist in achieving more stable complexes and in covering a larger conformational space of TCR-pMHC contacting interfaces. Nevertheless, in our more detailed research on TCR-peptide contacting residues, we used both repacked and minimally optimized models.

Validation of modeling approaches using reference PDB structures.

To validate the proposed mutation modeling approach and compare our results with corresponding models built using available TCR modeling services, we analyzed sequences of all CDR3 loops of ternary TCR-pMHC complexes available in the PDB database. All found sequences were pairwise aligned separately for TCR-alpha and TCR-beta chains. Analyzing the built alignments, we selected 27 CDR3 sequences that could be modeled by introducing up to 6 sequential substitutions starting from some other template structures presented in the PDB database. Hereinafter, the number of introduced amino acid substitutions will be referred to as the “mutation order”. The list of all selected CDR3 sequences with initial templates for the modeling process and their original PDB structures is presented in **Table SN1**

Table SN1 *Structures, used for validation of modeling approaches*

PDB template	TCR	CDR3	PDB original	Order
3qeq	TRA	CAFITGNQFYF	5d2l	2
5e6i	TRA	CAGAIGPSNTGKLIF	5euo	3
3qdm	TRA	CARNTGNQFYF	3gsn	2
1ao7	TRB	CASRPGLMSAQPEQYF	4ftv	4

5yxn	TRB	CASRRGSAELYF	5yxu	3
2vlr	TRB	CASSDTVSYEQYF	5nme, 5nmf, 5nmg	3
5nmg	TRB	CASSDWVSYEQYF	1lp9, 2j8u, 2jcc, 2uwe	1
6bj2	TRB	CASSFNMATGQYF	5brz, 5bs0	5
1oga	TRB	CASSIRSSYEQYF	5euo, 5hho	1
2vlj	TRB	CASSIRSSYEQYF	5euo, 5hho	1
5euo	TRB	CASSIRSSYEQYF	5hho	1
4l3e	TRB	CASSLSFGTEAFF	3qdg, 3qdj, 6am5, 6amu	1
5wkf	TRB	CASSPVTGGIYGYTF	3gsn	5
6avf	TRB	CASSQTQLWETQYF	5d2l	4
2vlj	TRB	CASSSRASYEQYF	2vlr	1
2vlr	TRB	CASSSRSSYEQYF	1oga, 2vlj, 2vlk, 2xn9, 4c56, 5hhm	1
5euo	TRB	CASSSRSSYEQYF	1oga, 2vlj, 2vlk, 2xn9, 4c56, 5hhm	1
4mji	TRB	CASSSWDTGELFF	3vxr, 3vxs	4
3qdg	TRB	CASSWSFGTEAFF	4l3e, 6d78, 6dkp	1
3vxr	TRB	CASSWSFGTEAFF	4l3e, 6d78, 6dkp	6

6amu	TRB	CASSWSFGTEAFF	4l3e, 6d78, 6dkp	1
2f53	TRB	CASSYLGNTGELFF	2p5e, 2p5w, 2pye	1
2p5w	TRB	CASSYVGNTGELFF	2bnq, 2bnr, 2f53, 2f54, 6q3s	1
4mnq	TRA	CAVDSATSGTYKYIF	4c56, 5men	4
5w1v	TRA	CAVLSSGGSNYKLTF	5eu6	4
5nqk	TRA	CAVSGGYQKVTF	2ypl	6
4g9f	TRA	CLVGEILDNFNKFYF	6avf	6

All T-cell receptor structures containing the selected CDR3 sequences were modeled using the TCRModel package of Rosetta software,TCRmodel2 and TCR-pMHC modeling service and our mutation approach. In addition to energy minimization and repacking of contacting residues, we performed a loop refinement procedure in our modeling approach as the final optimization step. Refinement was performed using Rosetta “LoopRemodel” mover with four “fixed” residues: two at each end of CDR3 loops.

As there can be some variability during conformational search and optimization of models, several runs of modeling were performed for each of the studied approaches: two runs of modeling were performed for our approach with different optimization procedures, two for the TCR-pMHC modeling service, three runs for TCRmodel, and two runs for TCRmodel2.

RMSD values between compared structures were calculated using the Profit application (Martin, A.C.R. and Porter, C.T., http://www.bioinf.org.uk/software/profit/, (5)) separately for all atoms of CDR3 loops and for C-alpha atoms only. During the study all models were superposed with original (target) PDBs by corresponding TCR chains or by CDR3 loops. Thus, RMSD values, calculated for structures aligned by CDR3 loops, represent the “accuracy” of predictions of corresponding loop shapes, while alignment using TCR chains allows the evaluation of both the loop shapes and their orientation. Results are presented in **Figure SN2**.

If modeled structures constructed based on one template had several known original target structures, mean values were calculated for the corresponding deviations. RMSD values of different modeling runs are presented independently on the graph.

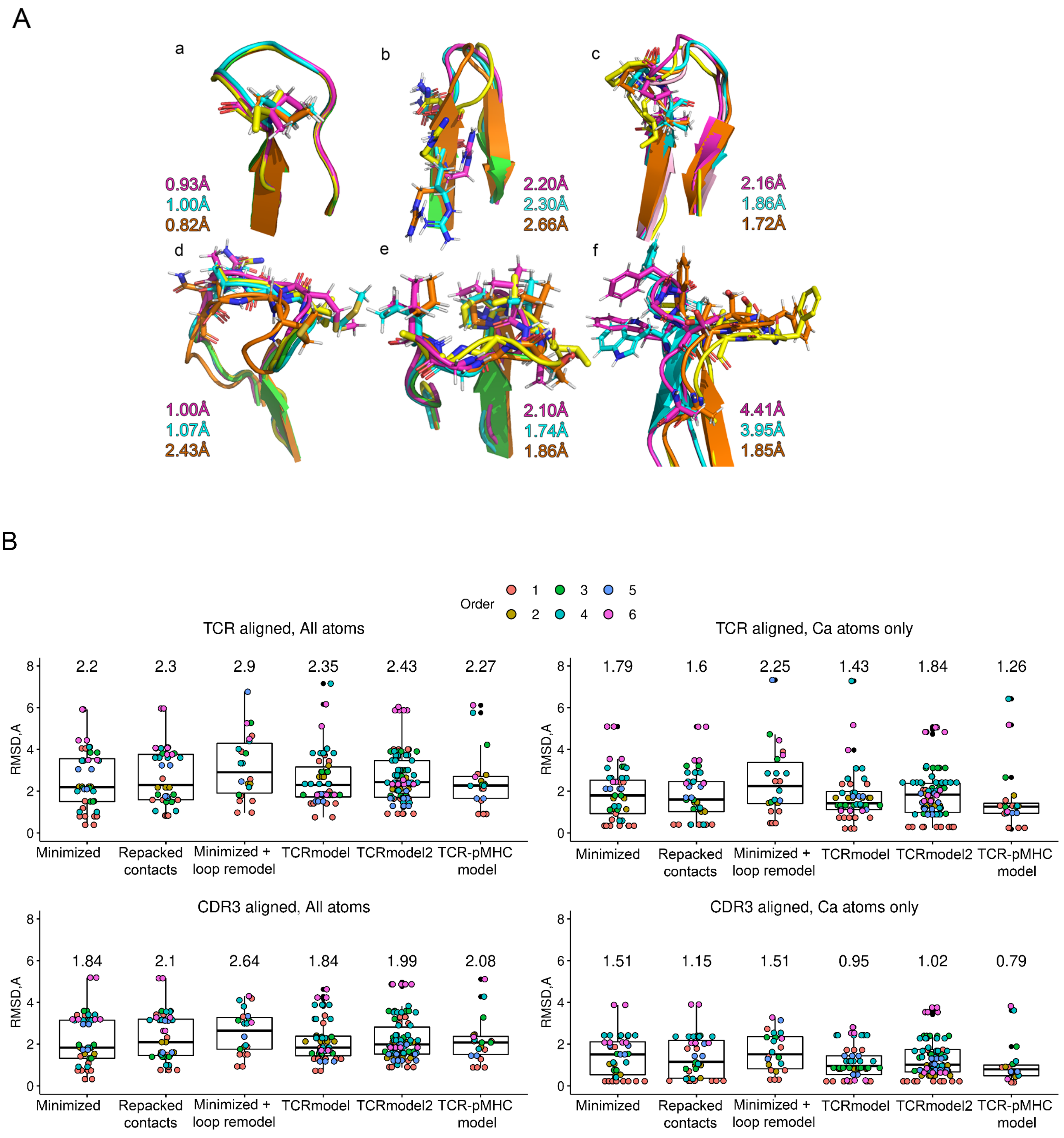

***Figure SN2** Comparison of modeled CDR3 structures with corresponding known conformations of CDR3 loops from PDB data bank.*

A) Structural comparison of models with different mutation orders. RMSD values, calculated for all atoms of CDR3 loops in structures aligned by TCR chains, are presented for three modeling approaches: our approach with energy minimization (magenta), our approach with repacking of contacting residues (cyan) and TCRmodel (orange). Initial template structures are colored green and original structures with target CDR3 loops are colored yellow. CDR3 sequences and mutation orders are as follows: a) Mutation order = 1, CDR3 sequence: CASSIRSSYEQYF (TRB, original structure- 5hho, 5euo; template- 1oga), b) Mutation order = 2, CDR3 sequence: CARNTGNQFYF (TRA, original structure- 3gsn; template - 3qdm), c) Mutation order = 3, CDR3 sequence: CAGAIGPSNTGKLIF (TRA, original structure- 5euo; template - 5e6i), d) Mutation order = 4, CDR3 sequence: CASRPGLMSAQPEQYF (TRB, original structure- 4ftv; template - 1ao7), e) Mutation order = 5, CDR3 sequence: CASSPVTGGIYGYTF (TRB, original structure- 3gsn; template – 5wkf), f) Mutation order = 6, CDR3 sequence: CASSWSFGTEAFF (TRB, original structure- 4l3e; template - 3vxr)

B) Boxplots of RMSD values between modeled CDR3 conformations and loops from original PDB structures. Median values for each modeling approach are presented in corresponding boxes. Models built using the mutation approach with finalizing energy minimization are labeled as "Minimized + loop remodel" and "Minimized" depending on whether loop remodel algorithm was applied or not. Models refined by repacking of all contacting residues between pMHC and TCR are labeled "Repacked contacts", and models built using TCRmodel, TCRmodel2 or TCR-pMHC modeling approaches are labeled "TCRmodel", "TCRmodel2" and "TCR-pMHC model" respectively.

Analysis of Cα atoms only in aligned CDR3 loops showed that all studied approaches allowed us to model conformations of the main chains of CDR3 loops close to the original structures. The median values for our approaches were 1.15Å for repacked structures and 1.51 Å for structures after just energy minimisation. The corresponding values for TCRmodel, TCRmodel2 and TCR-pMHC modeling services were 0.95Å, 1.02Å and 0.79Å.

Several outliers can be noted on the boxplots. These high RMSD values are associated with models carrying 6 substitutions (for all approaches) or 6-5 substitutions (for the TCR-pMHC model service).

As expected, median values of RMSD were higher during the analysis of full-atom structures of CDR3 loops including side-chain groups. The most significant increase was observed for structures modeled using the TCR-pMHC modeling service (from 0.79Å up to 2.08Å). The smallest median values were calculated for mutated structures with finalizing minimization and structures received using TCR-model (1.84Å for both mentioned approaches). Corresponding value for the TCRmodel2 was 1.99Å. CDR3 loops constructed using our approach with the loop refinement procedure at the final step had the lowest accuracy according to median RMSD values - 2.64Å during the analysis of structures aligned by CDR3 loops and 2.9Å for models aligned by corresponding TCR chains.

Analyzing all RMSD values, it can be observed that most of the modeled structures carrying from 4 up to 6 substitutions had lower accuracy than structures with mutation orders of 1-3. Median values of RMSD, calculated for structures carrying a different number of substitutions are presented in **Figure SN3**

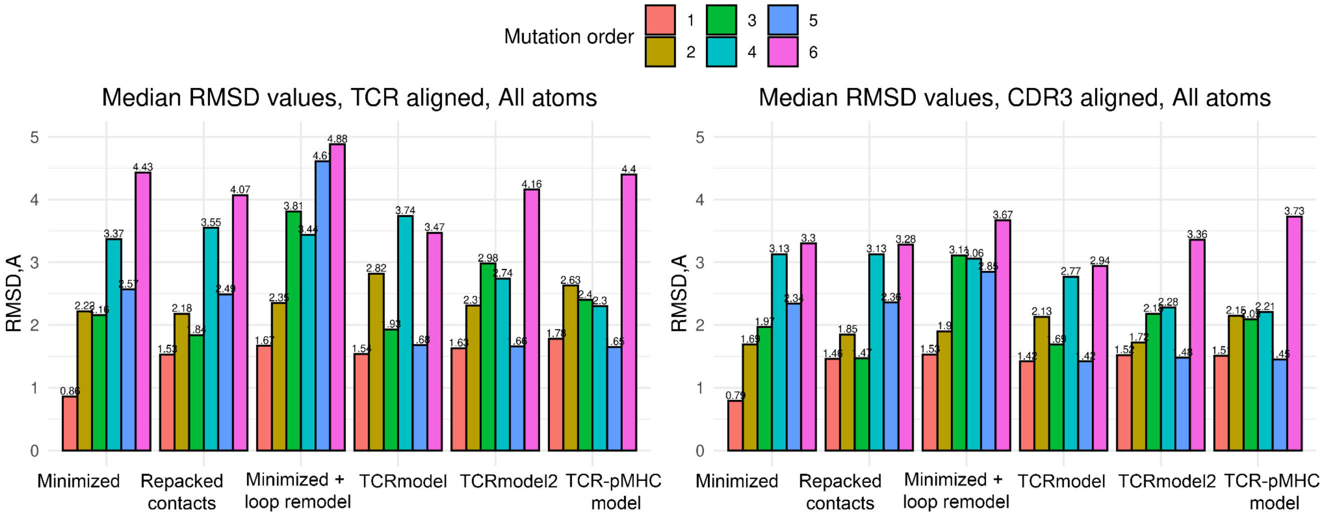

***Figure SN*3** *Median RMSD values, calculated for each mutation order individually*

It can be observed that the increase in RMSD values in all modeled structures correlated with the “mutation order”. According to the calculated RMSD values, the best models (closest to known structures) carried only one substitution and were built using our mutational protocol with only minimization as the final refinement step. The median values were 0.79Å and 0.86Å during the analysis of models aligned with the original structures by CDR3 loops and TCR chains, respectively. RMSD values for all other modeling approaches were in the range of 1.42Å -1.67 Å. The conformation and spatial orientation of CDR3 loops (**Figure SN3**, TCR aligned, all atoms), carrying two substitutions, were better predicted by our approaches with median RMSD values between 2.18Å and 2.35Å. TCRmodel, TCRmodel2 and TCR-pMHC modeling service predicted corresponding structures with 2.82Å, 2.31Å and 2.63Å deviation, respectively.

Third-order mutations were modeled with pretty close RMSD values of 2.16Å, 1.84Å using our approaches with minimization and repacking optimization. Loop remodeling, on the other hand, led to the increase of RMSD up to 3.81Å. TCRmodel, TCRmodel2 and TCR-pMHC modeling service predicted CDR3 loops structures with RMSD values of 1.93Å, 2.98Å and 2.4Å respectively.

According to median RMSD values of the models with mutation order = 4, the most accurate results were received using the TCRmodel2 and TCR-pMHC modeling service, while structures generated by TCRmodel had the RMSD value of 3.75Å. Also, it was noted that

TCRmodel, TCRmodel2 and TCR-pMHC modeling services predicted CDR3 loops carrying 5 sequential substitutions with 1.68, 166 and 1.65Å RMSD, that could be explained by their modeling algorithm: the mentioned services and approaches use (or had been trained on) their own templates database, that include target structures used in our benchmark.

This validation step showed that our approach with finalizing energy minimization or repacking of contacting residues in TCR - pMHC interface can be used to model CDR3 loops carrying up to 3 mutations with an acceptable accuracy. However, loop refinement, on the other hand, can decrease the quality of models with 1-3 mutation order.

Supplementary Note 1 References

1. Jensen KK, Rantos V, Jappe EC, Olsen TH, Jespersen MC, Jurtz V, et al. TCRpMHCmodels: Structural modelling of TCR-pMHC class I complexes. Sci Rep. 2019 Oct;9(1):14530. doi: 10.1038/s41598-019-50932-4

2. Gowthaman R, Pierce BG. TCRmodel: high resolution modeling of T cell receptors from sequence. Nucleic Acids Res. 2018 Feb;46(W1):W396–401. doi: 10.1093/nar/gky432

3. Yin R., Ribeiro-Filho HV, Lin V, Gowthaman R, Cheung M, Pierce B. TCRmodel2: high-resolution modeling of T cell receptor recognition using deep learning. Nucleic Acids Res. 2023 July; 51(W1):W569–W576. doi: 10.1093/nar/gkad356

4. Lindahl, Abraham, Hess, Spoel V der. GROMACS 2020 Manual [Internet]. 2020. Available from: https://zenodo.org/record/3562512

5. McLachlan AD. Rapid comparison of protein structures. Acta Crystallogr A. 1982 Jan;38(6):871–3. doi: 10.1107/S0567739482001806
